# Intratumoral immunotherapy with TLR7/8 agonist MEDI9197 modulates the tumor microenvironment leading to enhanced activity when combined with other immunotherapies

**DOI:** 10.1186/s40425-019-0724-8

**Published:** 2019-09-11

**Authors:** Stefanie R. Mullins, John P. Vasilakos, Katharina Deschler, Iwen Grigsby, Pete Gillis, Julius John, Matthew J. Elder, John Swales, Elina Timosenko, Zachary Cooper, Simon J. Dovedi, Andrew J. Leishman, Nadia Luheshi, James Elvecrog, Ashenafi Tilahun, Richard Goodwin, Ronald Herbst, Mark A. Tomai, Robert W. Wilkinson

**Affiliations:** 1 R&D Oncology, AstraZeneca Ltd, Aaron Klug Building, Granta Park, Cambridge, CB21 6GH UK; 2 3M Drug Delivery Systems Division, 3M Center Bldg 260-3A-14, St. Paul, MN 55144 USA; 3 R&D Oncology, AstraZeneca Ltd, 1 MedImmune Way, Gaithersburg, MD 20878 USA; 4 R&D Biopharmaceuticals, Pathology, Drug Safety and Metabolism, AstraZeneca Ltd, Cambridge, UK

**Keywords:** TLR, Immunotherapy, Immune checkpoint blockade, T cell agonist, T cell

## Abstract

**Background:**

Immune checkpoint blockade (ICB) promotes adaptive immunity and tumor regression in some cancer patients. However, in patients with immunologically “cold” tumors, tumor-resident innate immune cell activation may be required to prime an adaptive immune response and so exploit the full potential of ICB. Whilst Toll-like receptor (TLR) agonists have been used topically to successfully treat some superficial skin tumors, systemic TLR agonists have not been well-tolerated.

**Methods:**

The response of human immune cells to TLR7 and 8 agonism was measured in primary human immune cell assays. MEDI9197 (3M-052) was designed as a novel lipophilic TLR7/8 agonist that is retained at the injection site, limiting systemic exposure. Retention of the TLR7/8 agonist at the site of injection was demonstrated using quantitative whole-body autoradiography, HPLC-UV, and MALDI mass spectrometry imaging. Pharmacodynamic changes on T cells from TLR7/8 agonist treated B16-OVA tumors was assessed by histology, quantitative real time PCR, and flow cytometry. Combination activity of TLR7/8 agonism with immunotherapies was assessed in vitro by human DC-T cell MLR assay, and in vivo using multiple syngeneic mouse tumor models.

**Results:**

Targeting both TLR7 and 8 triggers an innate and adaptive immune response in primary human immune cells, exemplified by secretion of IFNα, IL-12 and IFNγ. In contrast, a STING or a TLR9 agonist primarily induces release of IFNα. We demonstrate that the TLR7/8 agonist, MEDI9197, is retained at the sight of injection with limited systemic exposure. This localized TLR7/8 agonism leads to Th1 polarization, enrichment and activation of natural killer (NK) and CD8^+^ T cells, and inhibition of tumor growth in multiple syngeneic models. The anti-tumor activity of this TLR7/8 agonist is enhanced when combined with T cell-targeted immunotherapies in pre-clinical models.

**Conclusion:**

Localized TLR7/8 agonism can enhance recruitment and activation of immune cells in tumors and polarize anti-tumor immunity towards a Th1 response. Moreover, we demonstrate that the anti-tumor effects of this TLR7/8 agonist can be enhanced through combination with checkpoint inhibitors and co-stimulatory agonists.

**Electronic supplementary material:**

The online version of this article (10.1186/s40425-019-0724-8) contains supplementary material, which is available to authorized users.

## Introduction

ICB monoclonal antibodies (mAbs) targeting inhibitory pathways at the T cell synapse to modulate T cell function have shown activity in the treatment of multiple cancers, as demonstrated by numerous regulatory approvals [1, 2]. However, monotherapy response rates to ICB mAbs remain low, and additional therapies are required. Clinical response to ICB mAbs correlates with the presence of high densities of tumor infiltrating CD8^+^ T cells (TILs, a so called “hot” tumor microenvironment) [3]. Patients with few TILs, so called “cold” tumors, tend to respond poorly to ICB mAbs. Therapeutic strategies that target tumor-resident innate immune cells and induce local tumor proinflammatory responses, which recruit CD8^+^ TILs, may be required to fully exploit the potential of ICB mAbs.

One strategy to turn an immunologically cold tumor hot is to promote activation of antigen presenting cells (APC) by targeting the endosomal TLRs TLR3, TLR7, TLR8, TLR9, or by targeting the ER-associated signalling molecule stimulator of interferon genes (STING). TLR3, TLR7, TLR8, and TLR9 recognise single and double stranded viral RNA and bacterial CpG DNA in the endosome following internalisation by APCs. STING senses aberrant DNA species or cyclic dinucleotides in the cytosol [4]. TLR and STING signalling activates APCs, increasing expression of inflammatory cytokines and co-stimulatory molecules, and enhancing antigen presentation capacity. Thus, APC activation by TLRs or STING can promote switching of CD4^+^ T cell response from Th2 to Th1, enhance CD8^+^ T cell responses, and inhibit T regulatory cell responses [5–9].

TLR7/8 agonists (imidazoquinolines), TLR9 agonists (synthetic CpG oligonucleotides) and STING agonists (cyclic di-nucleotides) have all shown potent anti-tumor activity in a range of murine cancer models [4]. The clinical utility of systemically delivered TLR agonists has been limited due to toxicity [10, 11]. In contrast, direct dermal application of Imiquimod (TLR7 agonist) or Resiquimod (TLR7/8 agonist) promotes local immune activation while limiting systemic exposure and side-effects and has clinical activity in treating multiple cutaneous tumor types [12]. Imiquimod is also approved as a topical treatment for external genital warts, actinic keratosis, and superficial basal cell carcinoma (Aldara approved product labelling; [12–16]). However, the utility of these topical treatments is confined to patients with cutaneous lesions*.*

Intratumoral (IT) TLR or STING agonist therapy has the potential to treat patients with solid tumors. The potential of IT therapeutic anti-cancer agents in the clinic is underscored by the recent approval of the oncolytic viral product T-VEC [17, 18]. In preclinical models, TLR and STING agonists delivered IT have promising anti-tumor activity [4, 19].

A TLR7/8 agonist has the potential to activate a broader range of human APCs versus targeting TLR9 within the tumor microenvironment, since TLR7 and TLR9 are both expressed on plasmacytoid dendritic cells (pDCs) and B cells, whereas TLR8 is more widely expressed on monocytes and myeloid dendritic cells (mDCs) [20]. Furthermore, it was hypothesized that retention of the TLR7/8 agonist in the tumor may be important for efficacy. Since the TLR7/8 agonist Resiquimod is known to distribute quickly into the systemic circulation after injection [21], MEDI9197, a dual TLR 7/8 agonist, was designed with a lipid tail to reduce aqueous solubility leading to retention at the injection site, rather than rapidly distributing throughout the body. MEDI9197 has been shown to have activity in mouse vaccine and cancer models and synergize with other immunotherapies [22–24].

In this manuscript we demonstrate that targeting TLR7 and 8 compared with TLR9 or STING in human PBMCs leads to induction of a broader range of cytokines. In murine syngeneic tumor models, MEDI9197 modulates the tumor microenvironment (TME) to an inflamed immunophenotype and increases the anti-tumor efficacy of immune co-stimulatory molecule agonists.

## Materials and methods

### TLR reporter assay

Human TLR7, human TLR8, or mouse TLR7 HEK293 reporter cells (InvivoGen) were seeded at 3 × 10^4^ cells/96-well for 24 h then treated with MEDI9197 or DMSO for 24 h. Secreted alkaline phosphatase (SEAP) was measured from supernatants using QuantiBlue reagent (InvivoGen).

### DC activation and cytokine production

pDC and mDC were enriched from PBMCs by magnetic bead negative selection (Miltenyi Biotec). Cells were resuspended at 4 × 10^5^ cells/mL in serum-free AIM V medium and cultured for 24 h in 96-well plates with MEDI9197 or DMSO. Supernatants were analysed for IFNα and IL-12p40 by ELISA (Mabtech and Affymetrix eBioscience, respectively).

### IL-12 release in human macrophages

CD14^+^ cells were purified from PBMCs using the EasySep™ Human Monocyte Enrichment Kit (StemCell Technologies) and differentiated into macrophages with 100 ng/mL recombinant human M-CSF (Peprotech). After 6 days cells were harvested, seeded in 96 well plates and rested overnight. Cells were primed with 20 ng/mL human recombinant IFNγ (R&D systems) for 24 h, followed by 3 μM MEDI9197 or 20 ng/mL LPS (Invivogen) for 24 h. Supernatants were analysed for IL-12p70 using the human IL-12p70 ELISA Duoset kit (R&D).

### PBMC activation, cytokine and FACS analysis

3–5 × 10^5^ PBMCs were seeded in 96-well plates and stimulated with MEDI9197 (in DMSO), CpG (ODN 2395 VacciGrade™ in H_2_O, Invivogen), STING agonist (2′3’-c-di-AM (PS)2 (Rp,Rp) VacciGrade™ in H_2_O, Invivogen), Resiquimod (in DMSO, Sigma-Aldrich), or Imiquimod (in DMSO, R&D Systems) at the indicated concentrations for 24 h at 37 °C. Flow cytometry was performed by standard procedures with intracellular fixation buffer from eBioscience. See Additional file 1 for a list of Flow cytometry antibodies. IFN-gamma was analysed by ELISA (Standard: human recombinant IFNγ (R & D). Capture antibody: Anti-human IFNγ (Clone NIB42, Pharmingen). Detection antibody: Biotinylated anti-human IFNγ (Clone 4S.B3, Pharmingen) or MSD (Mesoscale Diagnostics). IFN-alpha was analysed by ELISA (MABTECH) and IL-12p70 by MSD (Mesoscale Diagnostics). For ELISAs, DELFIA readout (Perkin Elmer) was used.

### PHA-induced cytokine assay

2 × 10^5^ human PBMCs were seeded in 96-well plates and treated with MEDI9197 or DMSO for 2.5 h, then incubated with 10 μg/mL PHA-L (Roche) for 45.5 h at 37 °C. IL-5 was assessed in supernatants using human IL-5 ELISA Set (BD OptEIA,) with DELFIA readout (Perkin Elmer).

### Natural killer (NK) cell killing assay

2 × 10^6^ cells/mL human PBMCs were cultured for 24 h at 37 °C with 3 μM MEDI9197 or DMSO. NK cells were then isolated using an EasySep® Human NK Cell Enrichment Kit (Stemcell). K562 target cells (ECACC,) were loaded for 20 min with DELFIA BATDA reagent (Perkin Elmer) at 37 °C, washed 5 times in PBS, 0.1% (v/v) BSA, 2.5 mM probenecid (ThermoFisher Scientific) and co-cultured with NK cells for 3 h at 37 °C in the presence of 2.5 mM probenecid. Killing was assessed in supernatants by DELFIA EuTDA readout (Perkin Elmer). Percent specific killing was calculated from the fluorescence values of ((Sample – (Target cells only – medium) – (NK cells only – medium) – medium) × 100)/(100% lysis – medium).

### Peptide-specific recall assay

2.5 × 10^5^/96-well of human PBMCs from donors pre-screened for reactivity against CMV peptide NLVPMVATV. were stimulated with 3 μM MEDI9197 or DMSO and a titration of CMV peptide. After 5 days, cells were stained for flow cytometry analysis. A list of antibodies and details of flow cytometry can be found in Additional file 1 section.

### Mice and rats

Female C57BL/6 J, BALB/cAnNCrl, C57BL/6 J-TyrC-2 J (C57BL/6 J Albino, Strain B6(Cg)-TyrC-2 J/J), CD-1® IGS mice (Crl:CD1(ICR)) (Charles River or Jackson Laboratories) at 6–10 weeks of age (~ 18–25 g) were used for in vivo studies. Male Sprague Dawley rats (Crl:CD (SD)), 200–400 g, were obtained from Charles River Laboratories.

### In vivo studies

The melanoma cell line B16-OVA was obtained from Dr. Wynette Dietz, University of Minnesota. Cell suspensions were implanted subcutaneously: 5 × 10^5^ B16-OVA cells, 5 × 10^3^ B16-F10 AP3 CAG luc2 (AstraZeneca) cells in 1:1 Matrigel (Corning)/PBS, or B16-F10 AP3, 4 T1 and MC38 cells as previously described [25].

Tumor volume = (Length × Width^2^)/2 = mm^3^. Mice were euthanized when tumors reached an average diameter of 15 mm (or 20 mm for B16-OVA tumors). Survival was defined as survival to a humane endpoint, based on tumor volume or average diameter and overall condition of the animal.

MEDI9197 and Resiquimod were prepared in sesame oil with 7.5% EtOH (w/w) and citrate buffer, respectively. The O/W formulations consisted of 74% (v/v) 10 mM NaCitrate, 20% (v/v) Soybean Oil, 3% (v/v) Tween 80, and 3% (v/v) Span 85 with or without 0.04% (w/w) MEDI9197 or Resiquimod.

MEDI9197, Resiquimod, or Vehicle solution were administered via IT or subcutaneous (SC) injection. Anti-mouse PD-L1: clone 80 mIgG1 D265A (AstraZeneca), anti-rat PD-L1: clone 10F.9G2 (BioXcell), Mouse GITRL fusion protein [26], anti-mouse OX40: Clone OX86 mIgG_2a_ (AstraZeneca), anti-mouse NK1.1: clone PK136 mIgG_2a_ (BioXcell) or corresponding isotype controls (NIP, AstraZeneca) were injected via intraperitoneal (IP) injection.

For TIL analysis (gene expression or flow cytometry) tumors collected from 6 days after MEDI9197 treatment were enriched for responders. Responders are defined as tumors with drug-induced tumor growth inhibition (average tumor volume < 600 mm^3^). 50% of MEDI9197 treated tumors were considered responders and groups were powered for this.

### Quantitation of [^14^C]-MEDI9197 in rat tissues, whole blood, and plasma

[^14^C]-MEDI9197 was quantified in rat tissues, whole-blood, and plasma by Quotient Bioresearch Ltd., UK after SC injection. Whole-blood and plasma samples were collected via a tail vein or via cardiac puncture. Plasma samples were taken for direct Quantitative Radiochemical analysis (QRA). Whole-blood samples were taken for oxidation prior to QRA.

Distribution of radioactivity in tissues was determined by quantitative whole-body autoradiography (QWBA). Tissue concentrations of radioactivity were determined using calibrated autoradiographic microscales (GE Healthcare). For calculation of the weight equivalent/g data, the nCi/g data was divided by the relevant specific activity (84.5 μCi/mg).

### TNFα quantitation from rat serum

TNFα was measured in undiluted serum using Invitrogen’s antibody bead kits, according the manufacturer’s instructions. The LLQ for TNFα is 2 pg/mL.

### MALDI mass spectrometry imaging

MALDI-MSI experiments were carried out in positive reflectron mode over a mass range of *m/z* 200 to 1000 using a MALDI rapifleX tissuetyper (Bruker Daltonics) equipped with a 10 kHz Smartbeam 3D™ Nd:YAG laser. Data collected on the rapifleX was at a spatial resolution of 50 μm, summing up 500 laser shots/raster position.

FlexImaging 5.0 (Bruker Daltonics) software was used for initial data analysis. Normalization, molecular image extraction and spectral clustering were defined in SCiLS Lab 2018b (Bruker Daltronics) software typically using mass selection window of ±0.05 Da. MEDI9197 and heme were detected at *m/z* 594.4 and 616.1, respectively.

### MEDI9197 quantitation from tumors and serum

MEDI9197 quantitation was performed as previously described [27].

### Optical imaging of tumor burden

Mice implanted with B16-F10 CAG luc2 were administered Xenolight D-Luciferin K^+^ salt bioluminescent substrate (IP, 100 μL of 33 mg/mL, PerkinElmer). 15 min after substrate injection, mice were imaged on an IVIS100 under isoflurane at an exposure time of 1 s using an open filter and field of view C. Image analysis was completed using living Image Software (PerkinElmer). Regions of interest were drawn around the tumors and total counts were generated within the region of interest.

### Tumor histology

Excised tumors were immersed in 10% buffered formalin and sent to Marshfield Lab, Marshfield, WI for paraffin embedding, sectioning, H&E staining, and histopathology evaluation. Digital photomicrographs were taken from all sections, and the number of lymphoid aggregates per section were quantified by microscopy (Veterinary Pathologist, Marshfield Lab).

### Tumor immune profiling by flow cytometry

Single cell suspensions from individual tumors were obtained using the murine tumor dissociation kit and a gentleMACS dissociator (Miltenyi Biotec). T and NK cells were stained with viability Zombie Aqua Dye (BioLegend) and fixed in 1% of paraformaldehyde at 4 °C for 30 min prior to FACS analysis.

For in vitro activation of TILs and measurement of intracellular cytokine producing T cells, leukocytes were enriched using anti-mouse CD45 MicroBeads and collected on LC columns using a MACS separator (Miltenyi Biotec). TILs were collected and resuspended in 1 mL TexMACSmedium (Miltenyi Biotec) containing cell stimulation cocktail plus protein transport inhibitors (eBioscience/ThermoFisher Scientific) for 16 h. Activated TILs were evaluated in staining buffer containing protein transport inhibitor cocktails (eBioscience/ThermoFisher Scientific) until the permeabilization/fixation step. A list of all FACS antibodies can be found in the Additional file 1 section.

### Measurement of gene expression in mouse tumors

Tumors were stored in RNAlater® RNA stabilization solution at − 20 °C (Life Technologies) then homogenized in RLT buffer using Miltenyi M tube with a gentleMACS dissociator. Total RNA was isolated using RNeasy Mini Kit according to the manufacturer’s protocol. gDNA was removed using RNase-free DNase I (Qiagen).

cDNA was generated using RT2 First Strand Kit (Qiagen). qPCR assays were carried out either using RT2 Profiler PCR Array (Qiagen, Cat. no. 330171 CLAM24673) or PrimeTime qPCR assays (Integrated DNA Technologies). Probe lists can be found in the Additional file 1 section. All qPCR assays were performed with a LightCycler 96 (Roche) using cycling conditions provided by assay manufacturer.

Relative expression levels were quantified using ΔCt (GusB Ct – Target gene Ct).

Fold-Change = 2^-ΔCt(Treated)^ ÷ 2^-ΔCt(Control)^ = Relative quantitation of treated (MEDI9197) ÷ Relative quantitation of Untreated (Vehicle).

### Measurement of tumor specific T cells by ELISpot

Tumors were excised at days 3, 7, and 11 post-dose (IT-20 μg MEDI9197 or IT-Vehicle). TILs were isolated from tumors as described above (Tumor immune profiling section). Lymphocytes from 1 to 3 animals were pooled and 20,000–100,000 lymphocytes per well were analyzed for IFNγ and Granzyme B expression using a dual color ELISpot assay (R&D Systems) following manufacturer’s protocol. Lymphocytes were stimulated in duplicate for 24 h in 200 μL AIM-V with PMA/Ionomycin following manufacturer’s instructions (BioLegend) or 5 μg/mL of a known H-2K^b^ [OVA (257–264):SIINFEKL, p53(232–240):CNSSCMGGM, TRP-2(181–188):SVYDFFVWL, MAGE-A5 [5–12]:HNTQYCNL, Sendai virus nucleoprotein (324–334) FAPGNYPAL] or H-2D^b^ [gp100 [25–33]:EGSRNQDWL and Influenza A nucleoprotein (366–374):ASNENMETM] peptide epitopes. SIINFEKL peptide was from InvivoGen all other peptides were from Genscript. All peptides were dissolved at 1 mg/mL in DMSO. ELISpot assay was performed following manufacturer’s instructions. Spots were counted using a CTL ImmunoSpot S6 Micro using ImmunoSpot 7.0 ProDC suite (CTL Analyzers, LLC).

### Human DC-T cell MLR primary cell assay

Human Mo-DCs were generated by differentiation of CD14^+^ monocytes (EasySep Human CD14+ positive selection kit (Stemcell)) in the presence of 100 ng/ml GM-CSF and 100 ng/mL IL-4 for 6 days in X-Vivo15 with 2% human AB serum. Mo-DCs were harvested, seeded, and stimulated with MEDI9197 for 18 h. Allogeneic human CD3^+^ T cells were isolated using the Human T cell enrichment kit (Stemcell), then added to the mo-DCs at a ratio of 10:1 and co-cultured for 3–5 days. IL-2 was measured by DELFIA ELISA (R&D) in day 3 supernatants and IFNγ (BD) in day 5 supernatants.

## Results

### MEDI9197 activates human innate and adaptive immune cells

**Fig. 1:**
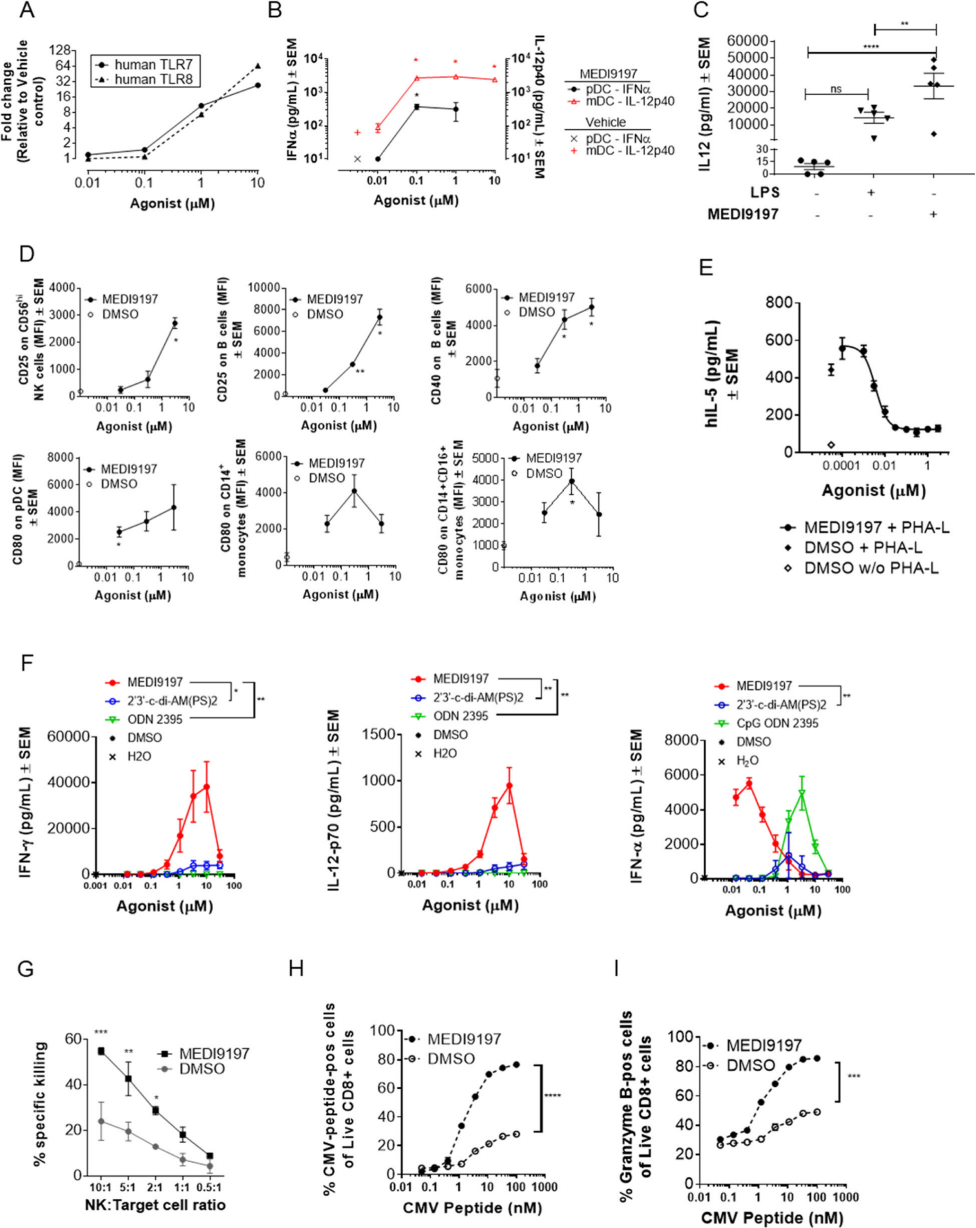
MEDI9197 activates innate and adaptive immune cells. **a** SEAP reporter activity in HEK293-NFκB-SEAP cells expressing human TLR7 or TLR8. Results are shown as fold-change relative to DMSO. **b** pDC and mDC enriched from peripheral blood and treated with MEDI9197 or DMSO. IFN-α and IL-12p40 secretion was measured from pDC and mDC, respectively, by ELISA. Results are shown as mean ± SEM, n = 3 donors. **c** IL-12p70 cytokine release from 20 ng/mL LPS or 3 μM MEDI9197-treated monocyte derived macrophages was tested by ELISA. Results show the mean of 5 donors. **d** Median fluorescence intensity (MFI) of activation markers on cell subsets from human PBMCs stimulated with MEDI9197 or DMSO. NK cells = CD3^−^ CD19^−^ CD56^high^; B cells = CD3^−^ CD19^+^; pDC = CD3^−^ CD19^−^ CD20^−^ CD56^−^ HLA-DR^+^ CD14^−^ CD16^−^ CD123^+^ BDCA4^+^; monocytes = CD3^−^ CD19^−^ CD20^−^ CD56^−^ HLA^−^DR^+^; Data show the mean ± SEM, representative of 4 donors. **e** IL-5 release (ELISA) from human PBMCs stimulated with MEDI9197 or DMSO with or without PHA-L. Data show mean of technical triplicates ±SEM, representative of 9 donors. **f** Cytokine production (ELISA and MSD) from human PBMCs stimulated with MEDI9197, C class CpG, or STING agonist. Results show mean ± SEM of 3 donors, representative of 6 donors. **g** Percent specific killing of Eu-loaded K562 target cells co-cultured with NK cells isolated from human PBMCs stimulated with MEDI9197 or DMSO. Data show mean of technical duplicates ±SEM, 9 donors. **h** and **i** Proportion of CMV peptide-specific CD8 cells (**h**) or granzyme B-positive CD8 cells (**i**) after stimulation of PBMCs with MEDI9197 or DMSO and a titration of CMV peptide, 2 donors. Data are representative of ≥2 independent experiments. Statistical analyses were performed by one-way (C) and two-way (F-I) ANOVA with Tukey’s (C, F, H, I) or Sidak’s (G) post hoc test or Welch’s upaired T test (one-tailed) (B, D). *p < 0.05, **p < 0.01, ***P < 0.001, ****p < 0.0001

MEDI9197 is a dual agonist for TLR7 and TLR8, as confirmed using HEK reporter cells transfected with human TLR7 or human TLR8 (Fig. 1a). Like other imidazoquinolines, MEDI9197 also activates murine TLR7, induces IFNγ from mouse splenocytes and has no negative impact on cell viability (Additional file 1: Figure S1A-C). Imidazoquinolines do not agonise murine TLR8, which may account for lower potency in mouse splenocytes compared with human PBMCs.

Given the broad expression of TLR7 and 8 on myeloid subsets [28] we investigated the impact of MEDI9197 on human myeloid cells. MEDI9197 induced IFNα and IL-12p40 release from pDC and mDC populations, respectively, which were enriched from human PBMCs **(**Fig. 1b). The ability of MEDI9197 to activate human DC populations was further demonstrated by increased IL-12p70 production from Monocyte-derived DCs (Mo-DC) (Additional file 1: Figure S1D). In human IFNγ-primed primary macrophages MEDI9197 induced IL-12p70 to significantly higher levels than a high dose of LPS (p = 0.0075; average of 33 vs. 14 ng/ml of IL-12 for MEDI9197 and LPS, respectively; Fig. 1c). This shift in macrophage polarization is further evidenced by upregulation of *CD40*, *CD80*, *IL12A* and *CD274 (PD-L1)* gene expression (Additional file 1: Figure S1E).

We next investigated the ability of MEDI9197 to stimulate innate and adaptive immune cells in human PBMC cultures. MEDI9197 induced the up-regulation of activation markers/co-stimulatory molecules from various human PBMC immune cell populations in vitro (Fig. 1d). These include: CD25 on NK and B cells; CD40 on B cells, and CD80 on pDCs, CD14^+^CD16^−^ monocytes, and CD14^+^CD16^+^ monocytes. Additionally, MEDI9197 skewed polyclonal immune responses away from a Th2-like phenotype by inhibiting IL-5 release from human PBMCs stimulated with PHA (Fig. 1e). When compared to a C-class CpG (ODN2395) or a STING agonist (2′3’-c-di-AM (PS)2) only MEDI9197 induced high levels of the Th1 cytokines, IFNγ and IL-12p70 from PBMC. The hook effect at high concentrations, which has been observed with other TLR7 agonists, is likely the result of differences in the rate of cytokine production [29] and is not due to cytotoxicity ([28], Additional file 1: Figure S1A). MEDI9197 also induced significantly greater IFNα production compared to treatment with the STING agonist and was more potent than C-class CpG. (p = 0.0013; Fig. 1f).

In addition to activating APCs, MEDI9197 enhanced the killing capacity of effector cells such as NK and T cells. Priming human PBMCs with MEDI9197 significantly increased target cell killing of K652 leukemia cells by isolated NK cells more than 2-fold at 10:1, 5:1 and even 2:1 NK:target cell ratio (p = 0.0001, p = 0.0013, p = 0.0185, respectively; Fig. 1g). In the context of CMV-specific PBMCs, priming with MEDI9197 and CMV-peptide led to an expansion of peptide-specific CD8^+^ T cells (Fig. 1h) with increased effector function (Granzyme B, Fig. 1i). Taken together these in vitro results show that MEDI9197 effectively stimulates the activation and function of innate and adaptive human immune cells.

### MEDI9197 is retained at the site of injection and induces pharmacodynamic responses

**Fig. 2:**
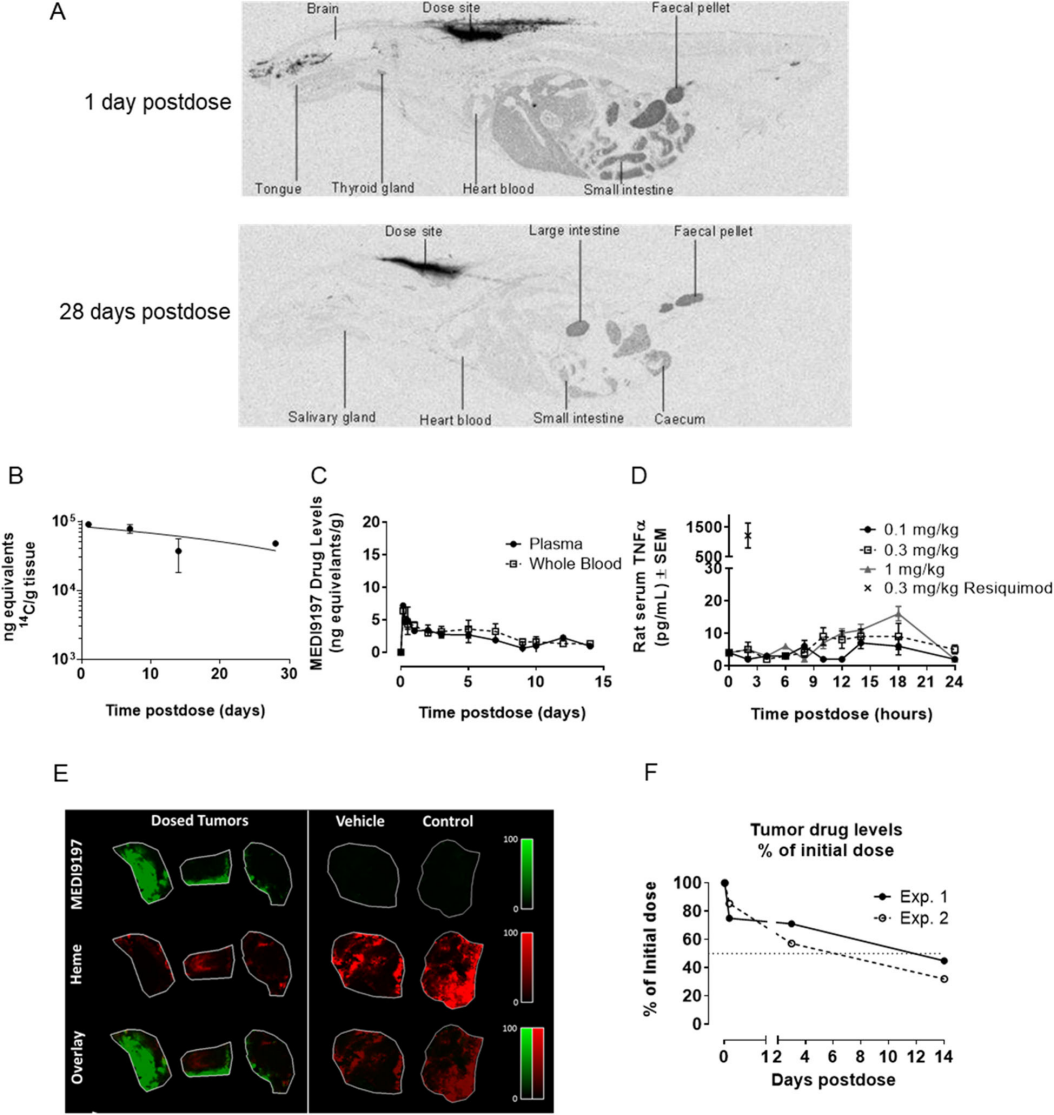
MEDI9197 is retained locally after injection. **a-c** Radiolabelled MEDI9197 distribution in male SD rats after SC injection. **a,** MEDI9197 distribution was measured in tissues by quantitative whole-body autoradiography 1 day and 28 days post-dose. Images of one rat serial transverse section (representative of two rats/time point). **b** and **c** Quantification of radiolabeled MEDI9197 at the injection site, plasma, and whole blood 4 to 672 h post-dose (mean, n = 2). **d** Rat serum TNFα levels following a single SC injection of 0.1, 0.3, and 1 mg/kg MEDI9197 or 0.3 mg/kg Resiquimod. The values at the 0 h time point are an average of 30 rats (1 serum sample per rat). The values shown at all other time points are an average of 5 rats (1 serum sample per rat). **e** MALDI-MSI images showing MEDI9197 and Heme distribution in B16-F10 AP3 tumors after a single 20 μg IT dose. Data are representative of 9 mice and 2 independent experiments. **f** Drug levels measured by HPLC-UV in B16-OVA tumors, implanted SC in C57BL/6-albino mice. After approximately 15 days, 50 μg MEDI9197 was injected IT, and tumors were collected immediately post-dose, 6 h, 3 days, and 14 days post-dose (5 mice/time point, 2 independent experiments). Tumor lysate drug levels are expressed as % of initial dose

The MEDI9197 lipid tail and formulation (sesame oil with 7.5% ethanol) have been designed to aid local retention of the TLR7/8 agonist at the site of injection to minimize systemic exposure and side effects, such as cytokine release syndrome (CRS). SC administration of radiolabelled MEDI9197 in rats revealed that MEDI9197 was retained at the site of injection for at least 28 days post dosing (Fig. 2a and b). Radiolabelled MEDI9197 was detectable in plasma and whole blood for at least 14 days (Fig. 2c). However, peak levels of MEDI9197 measured by LC-MS/MS in the serum from rats dosed SC or intramuscularly (IM) remained below 3 ng/mL (Additional file 1: Figure S2A, B). This is 20 times lower than the minimum effective concentration (MEC) of 59 ng/mL for MEDI9197 to induce cytokines from human PBMCs in vitro (data not shown). Low systemic exposure and subsequent pharmacodynamic (PD) effects of MEDI9197 were examined further in a separate rat study. MEDI9197 induced very low levels of systemic serum TNFα levels (peaking at 16 pg/mL 18 h post-dose) compared with Resiquimod (1212 pg/mL 2 h post-dose) after SC dosing (Fig. 2d).

MEDI9197 retention at the site of injection was qualitatively observed in the B16-F10 AP3 mouse melanoma model following administration of 20 μg MEDI9197 injected IT, by mass spectrometry (MS) imaging. MEDI9197 location in a section of excised tumor, indicated in green, was still detectable in concentrated regions of the tumor for at least 8 days post injection (Fig. 2e). The Heme signal is a measure of blood in the tissue slice, related to tissue damage, such as haemorrhage or necrosis. The signal for MEDI9197 does not colocalise with the MS signature for Heme (shown in red), therefore it is unlikely to be localized to a non-viable region of tissue. To determine the time-course of drug retention in B16-F10 tumors, MEDI9197 was quantified in homogenized tumors using HPLC-UV 6 h to 14 days after injection revealing that approximately 50% (indicated by dotted line) of the initial dose was retained in the tumor for approximately 9 days post injection (Fig. 2f). These data demonstrate that MEDI9197 is retained at the site of administration for up to 4 weeks after injection, with low systemic exposure.

To further evaluate the immunological effects of MEDI9197 retention at the injection site, naïve mice were SC injected with MEDI9197 or Resiquimod. Expression of TNFα, IL-12p40 and IFNγ mRNA was measured in the axilliary and brachial lymph nodes (dLN) proximal to the injection site (Additional file 1: Figure S3). The local dLN response was delayed with MEDI9197 (Tmax 6 h) compared to Resiquimod (Tmax 1 h). In the spleen, Resiquimod also induced rapid (Tmax 1 h) upregulation of these cytokine transcripts. MEDI9197, however, induced minimal changes in splenic TNFα, IL-12p40 and IFNγ expression after SC administration (Additional file 1: Figure S3). Thus, local retention of MEDI9197 minimizes distal inflammatory cytokine induction. SC injection of either MEDI9197 or Resiquimod induced expression of type I IFN response related genes (MX1, OAS2, Tnfs10) both in the dLN as well as peripherally in the spleen. However, the Tmax of gene expression for MEDI9197 dosed mice was 6 h, compared with 1 h for Resiquimod dosed mice (Additional file 1: Figure S3). Overall, these results show that retention of MEDI9197 at the injection site leads to a prolonged localized immune response with minimal systemic exposure or inflammatory cytokine expression.

### MEDI9197 induces a range of immunological changes within the tumor microenvironment (TME) resulting in anti-tumor efficacy

**Fig. 3:**
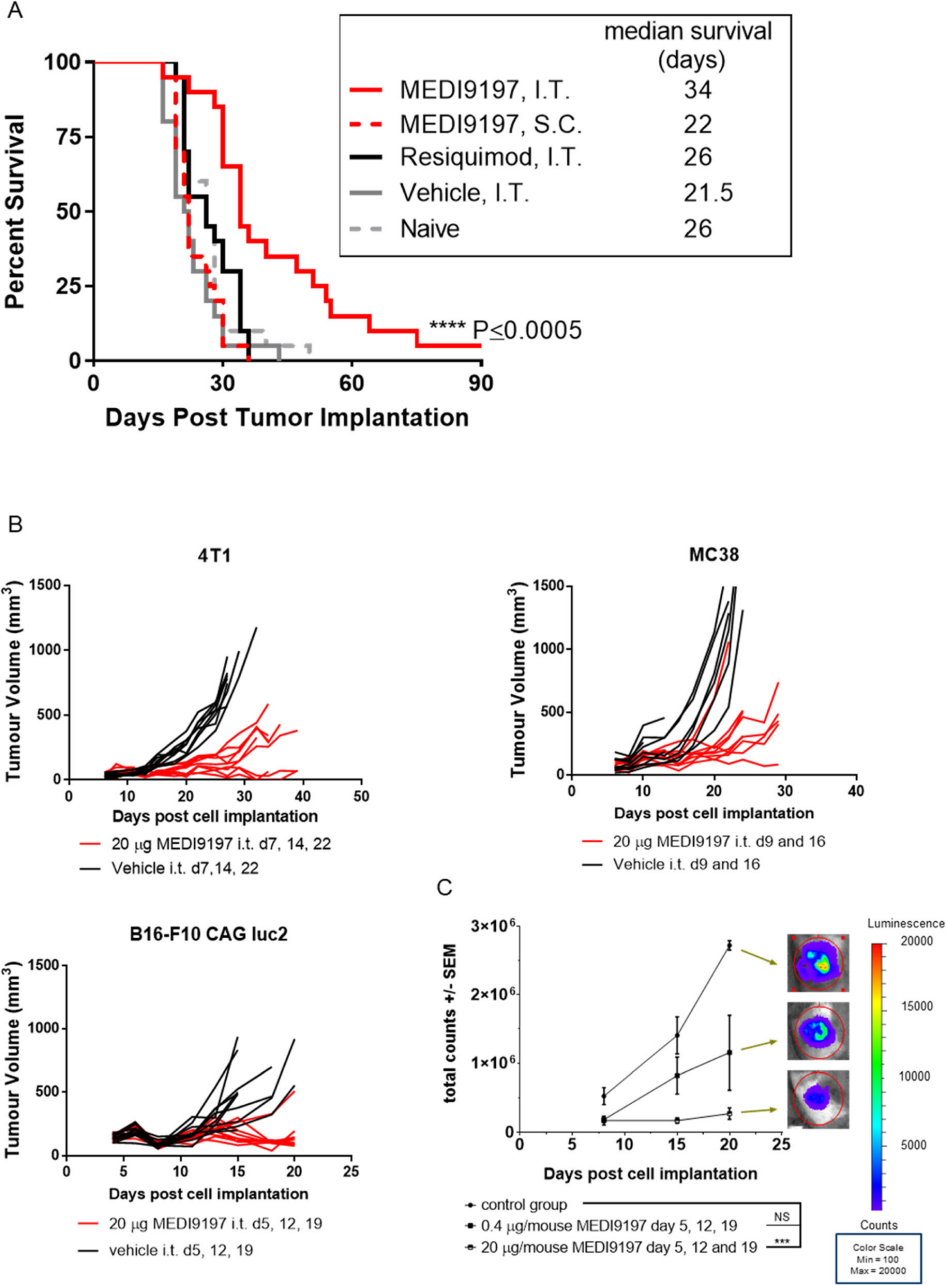
Intratumoral administration is required for MEDI9197 anti-tumor effects and is efficacious in diverse syngeneic models. **a** Anti-tumor effects was measured in the B16-OVA tumor model following IT injection or SC dosing, away from the tumor on the opposite flank. C57BL/6 J albino mice were implanted SC B16-OVA tumor cells the left flank on Day 0 (20 mice/group). On Days 8 and 15, mice were dosed IT with MEDI9197 [20 μg/50 μL], Resiquimod [20 μg/50 μL], or Vehicle (sesame oil/EtOH, 50 μL]). Some mice were dosed SC with MEDI9197 on the opposite side of the implanted tumor (right flank). Naïve mice were untreated. Mice were euthanized when tumor size equaled or exceeded 2500 mm^3^. The Kaplan-Meier plot shows survival for each group up to Day 90. ****P < 0.0005; MEDI9197 IT group (solid red line) compared with each of the other groups using the Log-rank test. **b** and **c**. Tumor volume was assessed in 4 T1, B16-F10 AP3 CAG luc2, MC38 single-flank mouse models following IT administration of MEDI9197 (20 μg) or Vehicle. MEDI9197 and Vehicle were dosed weekly for 2 or 3 doses as described in the figure. **b** Spider plots represent tumor volume of individual mice. Vehicle-treated mice are represented with black lines, and MEDI9197-treated mice are represented with red lines. **c** B16-F10 CAG luc2 tumors (9 mice/group) were treated with either 0.4 or 20 μg MEDI9197 weekly on days 5, 12, and 19. D-Luciferin was I.P. injected 15 min prior to imaging bioluminescence on the IVIS100. Data represents the total counts +/− SEM. Statistical analysis was performed by Dunnet’s multiple comparisons test. ***p = 0.0004, NS p = 0.1175. Representative images from day 20 post implantation are shown

Next, we investigated the effects of MEDI9197 on the TME and growth. MEDI9197, but not Resiquimod, administered IT, on days 8 and 15 post tumor cell implantation, significantly inhibited tumor growth and enhanced long-term survival by 12 days (p ≤ 0.0005) in mice bearing established B16-OVA melanoma tumors (Fig. 3a and Additional file 1: Figure S4a). When MEDI9197 was administered to mice SC, on the contralateral flank of the B16-OVA tumor, it was ineffective in mediating tumor control. Therefore, administration and retention of MEDI9197 at the tumor site is required for anti-tumor activity. Repeat dosing or doses higher than 20 μg administered IT did not further enhance anti-tumor activity (Additional file 1: Figure S4B-D). Furthermore, we show that IT administration of MEDI9197 can inhibit tumor growth in tumors previously described [25] to have: high (MC38); low (B16-F10 CAG luc2); and suppressive (4 T1) immune cell infiltrates (Fig. 3b). This highlights the potential of IT MEDI9197 to alter a wide range of TMEs to promote anti-tumor immunity. Additionally, imaging of luciferase expression in the B16-F10 CAG luc2 cells as an indicator of tumor burden demonstrates that MEDI9197 is effective at doses of 0.4 or 20 μg (Fig. 3c).

**Fig. 4:**
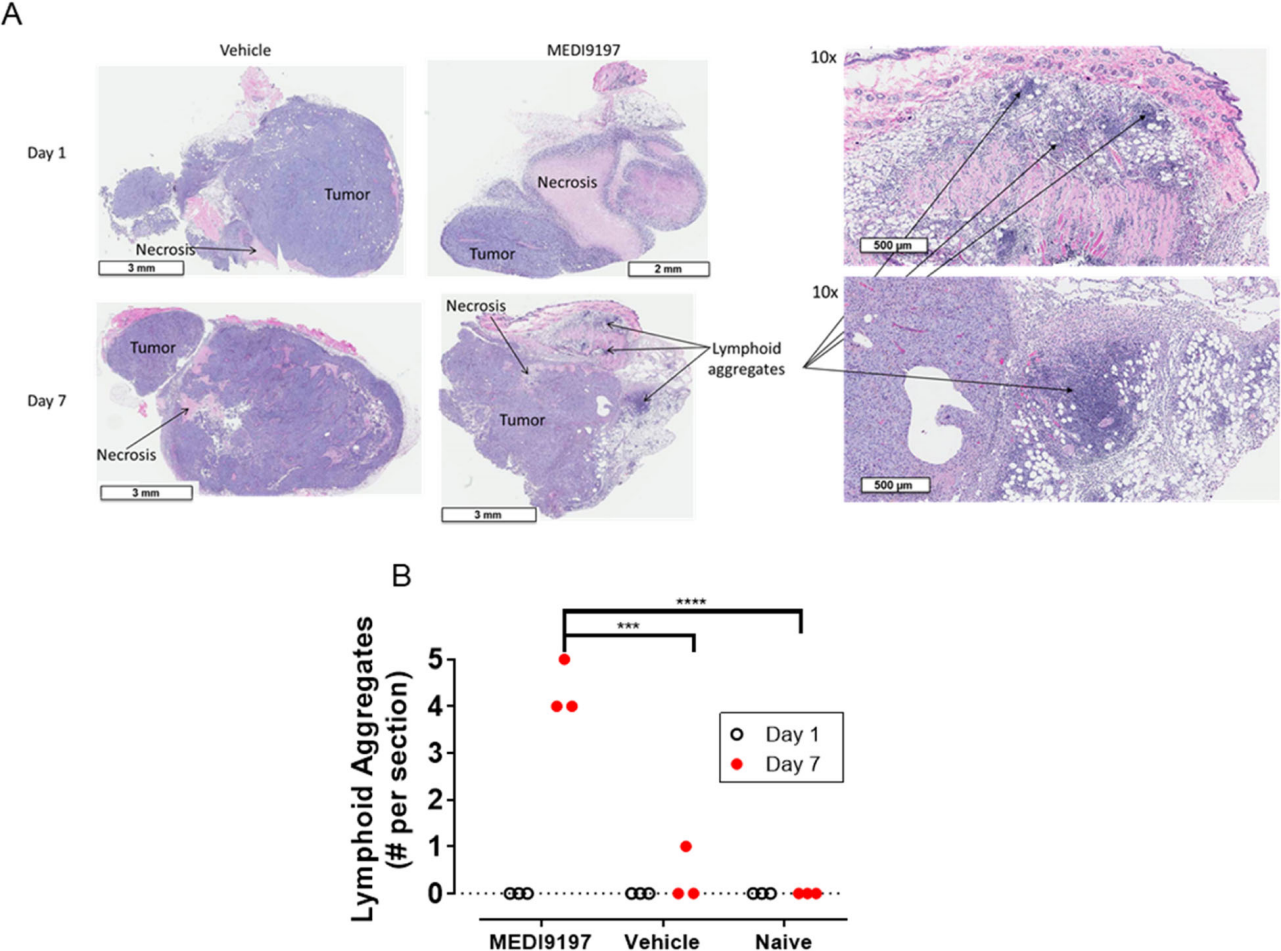
IT administration of MEDI9197 induces lymphoid aggregates in the tumor. **a** Gross histological changes to tumors dosed with MEDI9197 were evaluated in H&E stained tissue sections. Eight days after B16-OVA tumors were implanted SC into C57BL/6 J albino mice (3/group), a single dose of 20 μg MEDI9197 or Vehicle was administered IT. Some mice were untreated (Naïve, n = 3). After 1- and 7-days post-dose, FFPE tumors were sectioned, H&E stained, and scored for lymphoid aggregates. Each tumor photomicrograph is from one mouse and is representative of each treatment group. Data is representative of 3 independent experiments in the B16-OVA model. **b** Number of lymphoid aggregates per section for each mouse from each group is shown 1- and 7-days post-dose. Statistical analysis was performed by two-way ANOVA with Tukey’s post hoc test. ***p = 0.001, ****p < 0.001

Since localization of MEDI9197 in the TME is required for anti-tumor activity, we sought to test the hypothesis that MEDI9197 induces local conversion of the tumor immune infiltrate to promote anti-tumor immunity. Histological examination of B16-OVA tumors, 7 days after MEDI9197 treatment, revealed formation of lymphoid aggregates (ectopic lymph node-like structure) (Fig. 4a and b) indicative of localised immune responses. In comparison, Vehicle treated or naïve tumors had minimal or no lymphoid aggregates (Fig. 4b).

Gene expression analysis of MEDI9197 treated tumors compared with Vehicle treated tumors showed a strong upregulation of immune related genes in a subset of tumors treated with MEDI9197 (Additional file 1: Figure S5). At 7 and 11 days post-dose, a cohort of mice could be separated based on response to MEDI9197 treatment by tumor size compared with the Vehicle group. Those mice showing drug-induced tumor growth inhibition (tumour volume < 600 mm^3^, Responders) also correlated with the subset of tumors with a strong upregulation of immune related genes in contrast to those with no tumor growth inhibition which were similar to Vehicle control group, Non-responders. To better understand the mechanism of action for MEDI9197 anti-tumor activity, tumors that are responding to MEDI9197 treatment, based on tumor size compared with Vehicle, were used for pharmacodynamic analyses, where possible.

**Fig. 5:**
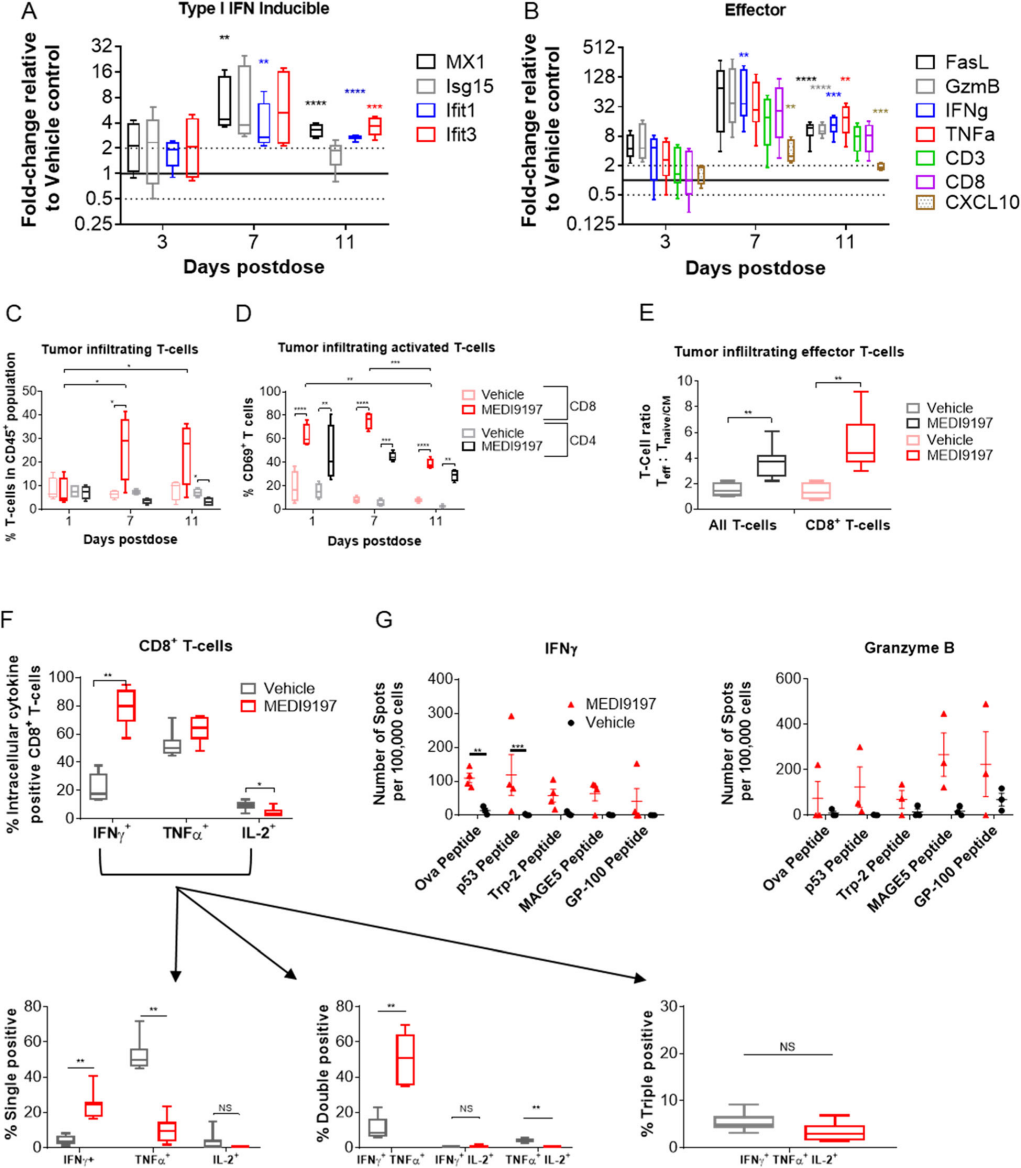
IT administration of MEDI9197 induces an increase in immune cell activation. SC implanted B16-OVA tumors were injected IT with 20 μg of MEDI9197 or Vehicle. The data for all graphs (except G) are shown in box-whiskers plots. Data is representative of at least 2 independent experiments. **a** and **b** Tumors were collected for qPCR analysis (performed in duplicate) (n = 5 per group). The data indicate fold-change in gene expression, relative to Vehicle, associated with type I IFN inducible (A) or T cell effector (B) genes. **c-d,** Percentage of T cells (CD3ε^+^ / CD4^+^ or CD8α^+^) in the CD45^+^ population and of activated T cells (CD69^+^) assessed by flow cytometry in individual tumors after MEDI9197 and Vehicle dosing (n = 4 mice/treatment group/day). **e** Ratio of effector T cells (T^eff^: CD45^+^/TCRβ^+^/CD44^+^/CD62L^−^) and naïve/central memory T cells (T^naive/CM^: CD45^+^/TCRβ^+^/CD44^+/−^/CD62L^+^) for total T cells and CD8^+^ cells (CD45^+^, TCRβ^+^, CD8α^+^), measured by flow cytometry in tumors injected with MEDI9197 (n = 11) or Vehicle (n = 7) and collected 6–8 days post-dose. **f** The top graph shows the percentage of intracellular cytokines in CD8α^+^ T cells (IFNγ, TNFα, IL-2, or negative), the three graphs below show the percentage of single, double, and triple positive CD8α^+^ T cells from tumors collected 4–5 days post dosing with MEDI9197 (n = 7) and Vehicle (n = 6). Cytokines were analyzed by flow cytometry in PMA/ionomycin stimulated CD45^+^ enriched cells. **g** TILs were isolated from each tumor 11 days post-dose (n = 3–4/group) and used for ELISpot analysis for IFNγ and Granzyme B following stimulation with PMA/Ionomycin or class I-restricted peptides (H-2K^b^ or H-2D^b^). The results show the number of spots per 100,000 cells after background subtraction based on control peptide (H-2D^b^ and H-2K^b^) stimulated TILs. Statistical analyses were performed using multiple T tests of dCT values (a, b), 2-way ANOVA (c, d, and g) with Sidak’s multiple comparisons test (c, d), and the Mann Whitney test (e, f). *p < 0.05, **p < 0.01, ***p < 0.001, ****p < 0.0001

We went on to characterize the changes in the immune phenotype of the TME. MEDI9197 treatment quickly induces a type I IFN response demonstrated by an increase in IFN-inducible genes (e.g. Mx1, Isg15, Ifit1 and Ifit3, Fig. 5a), which were increased at 3 days and peaked at 7 days post-dose. Expression of CD8^+^ T cell response genes (e.g. FasL, GzmB, and IFNg, Fig. 5b) peaked at 7 days post-dose and remained elevated at 11 days post-dose (e.g. IFNγ was approximately 32-fold and 15-fold higher than Vehicle control, respectively). Furthermore, the proportion of TILs made up by CD8^+^ T cells significantly increased 7 days and 11 days after MEDI9197 treatment (p = 0.0132 and p = 0.0589, respectively, compared with vehicle treatment), whereas the relative abundance of CD4^+^ T cells was decreased 11 days post-dose (Fig. 5c, Additional file 1: Figure S6B). Additionally, MEDI9197 enhanced activation of both CD4^+^ and CD8^+^ T cells at all timepoints tested (increased %CD69^+^ cells observed 1 day, 7 day and 11 days post-dose, Fig. 5d**,** Additional file 1: Figure S6B). NK cell activation in the tumor followed the same kinetics as T cell activation, but unlike CD8^+^ T cells, MEDI9197 did not increase the proportion of NK cells in the tumor (Additional file 1: Figure S6A and B). Overall, these results indicate that a single IT dose of MEDI9197 induces T cell and NK cell activation beginning 1 day after treatment and lasting at least 11 days post-dose and drives a significant increase in the relative abundance of CD8^+^ T cells at later timepoints.

We have observed that MEDI9197 can enhance human NK cell killing of target cells (Fig. 1) and can induce NK cell activation in vivo (Additional file 1: Figure S6A), whereas Singh et al. [23] have previously demonstrated that MEDI9197 anti-tumor activity is dependent on CD8^+^ T cells, but not NK cells, using depletion studies in the B16-OVA model, suggesting NK cells may be involved but are not required for the anti-tumor activity observed in the B16-OVA model, so we focused on further evaluation of the functional status of TILs. MEDI9197 induced an increase in total effector T cells (ratio of T^eff^ (CD45^+^/TCRβ^+^/CD44^+^/CD62L^−^) to T^naive/CM^ (CD45^+^/TCRβ^+^/CD44^+/−^/CD62L^+^) and effector CD8^+^ T cells (ratio of CD45^+^/TCRβ^+^/CD8α^+^/CD44^+^/CD62L^−^ to T^naive/CM^) in the tumor 6–8 days after dosing (Fig. 5e, Additional file 1: Fig. 6e**)**. In keeping with the induction of IFNγ gene expression in the TME detected by qPCR 7 days post-dose (Fig. 5b), MEDI9197 dosing induced a significant increase in the proportion of CD8^+^ T cells expressing IFNγ compared to the Vehicle group (p < 0.0001; 79.8% versus 17.6%) following ex vivo stimulation with PMA/ionomycin (Fig. 5f, Additional file 1: Figure S6D). The lower panels in Fig. 5f indicate that MEDI9197 induces significantly more double-positive (IFNγ/TNFα) CD8^+^ T cells compared to the Vehicle group (p = 0.0041; approximately 60% versus 10%). In contrast, most (about 70%) of the Vehicle-treated CD8^+^ T cells were single-positive (TNFα). There was no significant difference in the percentage of triple-positive (IFNγ/TNFα/IL-2) CD8^+^ T cells with MEDI9197 compared with Vehicle treatment. Eleven days post-dose, T cells from the tumor were responsive to multiple tumor associated antigens as demonstrated by IFNγ and granzyme B release after ex vivo stimulation with OVA or melanoma associated peptides (p53, TRP-2, gp100 and MAGE-A5) (Fig. 5g). These data indicate MEDI9197 induced upregulation of type I IFN response, IFNγ, and CD8^+^ T cell activation, all indicators of conversion to a hot TME favourable for anti-tumor immunity.

**Fig. 6:**
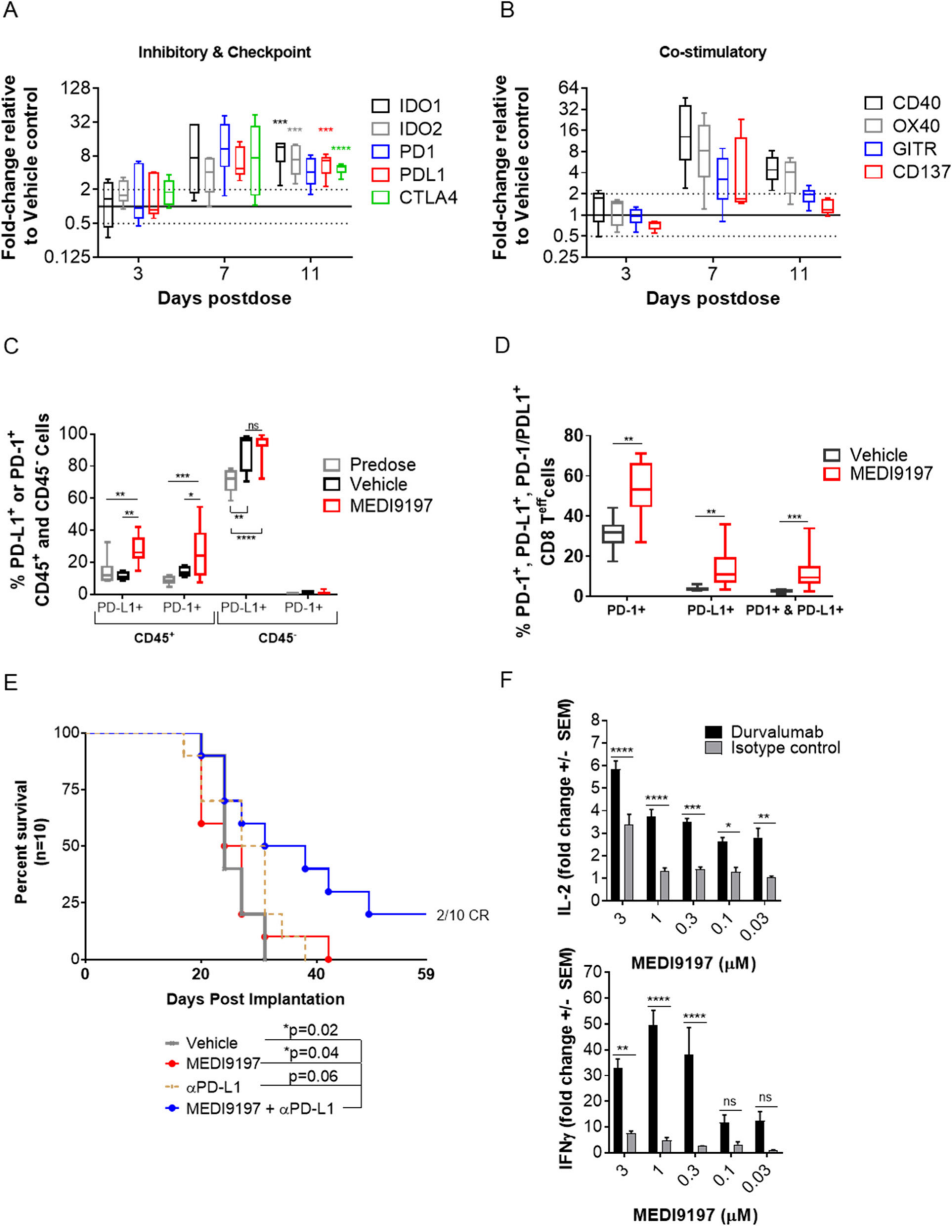
MEDI9197 enhances expression of PD1/L1 and increases immune stimulatory and anti-tumor effects of PD-L1 blockade. **a** and **b** The tumor immune profile was evaluated by qPCR following IT dosing. B16-OVA tumors were collected 3, 7, and 11 days post IT dose (20 μg MEDI9197 or Vehicle, n = 5). Total RNA was processed from each tumor, as described in Fig. 5a. The data indicate fold-change in gene expression associated with (**a**) Inhibitory and Checkpoint, and (**b**) Co-stimulatory genes. Fold-change is relative to the Vehicle group. Day 6–11 post MEDI9197 treatment results are enriched for responders. Multiple T tests were performed using dCT values to compare MEDI9197 treated versus Vehicle group (**a, b**). **c-d** Percentage of PD-1^+^ and/or PD-L1^+^ populations from tumor cells, TILs (CD45^+^) (**c**), and effector CD8α^+^ cells (CD45^+^/TCRβ^+^/CD8α^+^/CD44^+^/CD62L^−^) (**d**) measured by flow cytometry in tumors 6–8 days post-dose of MEDI9197 (n = 11) or Vehicle (n = 7). Statistical analysis used 2-way ANOVA with Tukey’s multiple comparisons test (**c**) and Mann-Whitney test (unpaired, non-parametric t test) (**d**). Data is representative of at least two independent experiments. **e** Kaplan-Meier plots of survival in the B16-OVA tumor model following MEDI9197 and anti-PD-L1 treatment. C57BL/6 albino mice (n = 10/group) were implanted SC in the right flank with B16-OVA tumors on day 0. On day 10, mice were dosed IT with 20 μg MEDI9197 or Vehicle, and twice weekly for 6 doses IP with 200 μg of anti-PD-L1 Ab or isotype control. CR, complete responder. Statistical analysis was performed using the Log-rank (Mantel-Cox) test. **f** Cytokine production after 3 (IL-2) and 5 (IFNγ) days of co-culture of allogeneic T cells with human Mo-DCs (10:1 ratio). DCs were primed for 18 h with a titration of MEDI9197 before medium change and addition of T cells plus 100 nM Durvalumab or NIP228 isotype control. Data are representative of 4 donors and statistical analysis was performed using two-way ANOVA with Bonferroni post-test. *p < 0.05, **p < 0.01, ***p < 0.001, ****p < 0.0001

### MEDI9197 enhances activity of IO therapies

The robust localized activation of immune cells and further recruitment of CD8^+^ TILs following MEDI9197 IT dosing indicates that this approach may compliment other IO therapies. We found that MEDI9197 induces an increase in gene expression for immune inhibitory (e.g. PD-1, PD-L1) and T cell co-stimulatory molecules (e.g. GITR and OX40) in the injected tumor (Fig. 6a and b). Based on the ability of MEDI9197 to induce CD8^+^ T cell activation and IFNγ production, as well as the increase in PD-1 and PD-L1 gene expression observed in the tumor after MEDI9197 treatment (Fig. 6a), it is not surprising that MEDI9197 also induced a significant increase in surface PD-1 expression on TILs, especially CD8^+^ T cells (p < 0.05 on CD45^+^ cells and p = 0.0059 on CD8^+^ effector T cells; Fig. 6c and d**,** Additional file 1: Figure S6C). MEDI9197 also induced a significant increase in PD-L1 on TILs including CD8^+^ T cells (p < 0.01 on CD45^+^ cells and p = 0.0012 on CD8^+^ effector T cells). Although a greater proportion of CD8^+^ T cells in the MEDI9197 group expressed cell surface PD-1 compared to PD-L1 (50% versus 10%), there were more CD8^+^ T cells in MEDI9197-treated tumors that expressed both cell surface PD-1 and PD-L1 (about 10% compared to 1% in the Vehicle group).

Given the increase in co-stimulatory or inhibitory molecules following IT dosing with MEDI9197 we explored whether combining MEDI9197 with T cell targeted therapies would result in enhanced anti-tumor efficacy. Similar to previous reports in the B16-F10 model [23], we found that MEDI9197 increases median survival observed with anti-PD-L1 mAb in the B16-OVA model from 29 to 34.5 days (Fig. 6**e**) and resulted in 2 out of 10 tumors regressing without regrowth for 59 days post tumor cell implantation, so we extended these findings to a human co-culture in vitro assay. Using a primary human DC-T cell mixed lymphocyte reaction (MLR) assay, we show that combining a PD-L1 blocking mAb (MEDI4736, durvalumab) with MEDI9197 increased IL-2 and IFNγ cytokine production versus MEDI9197 alone (Fig. 6f).

**Fig. 7:**
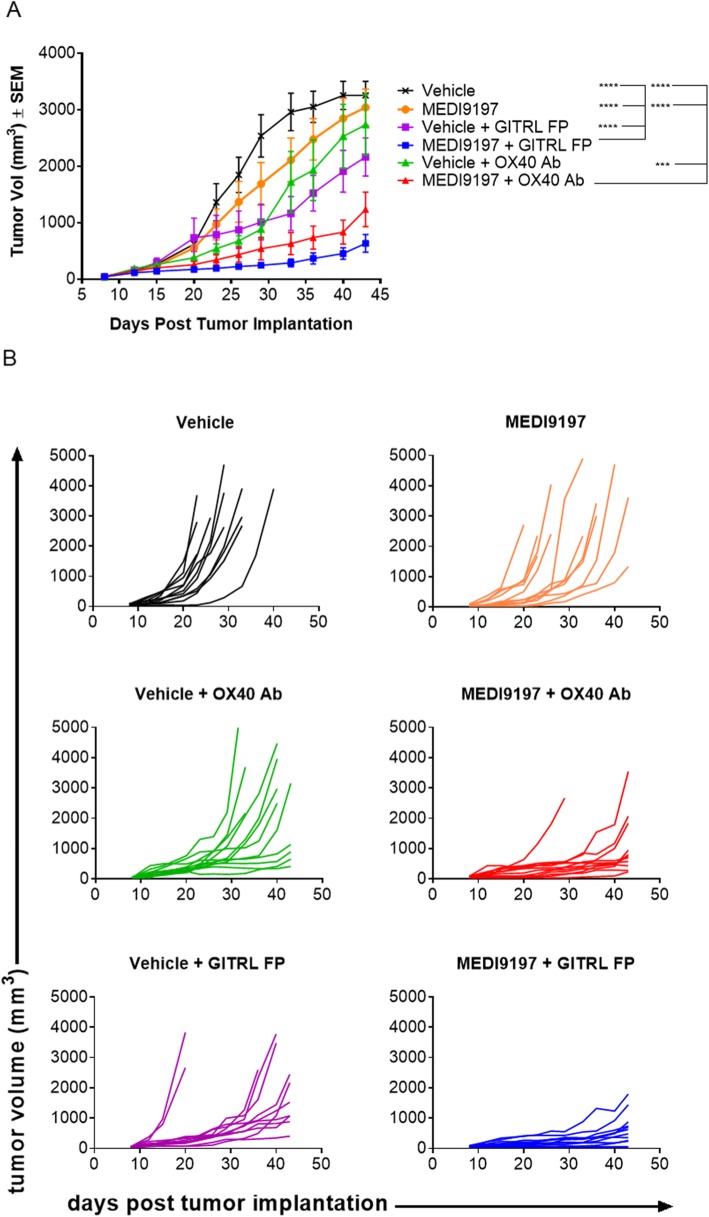
MEDI9197 enhances efficacy when combined with I-O agents targeting OX40 or GITR. Tumor growth was measured in the single-flank B16-OVA tumor model following IT injection of MEDI9197 and IP dosing with GITRL FP or OX40 antibody. C57BL/6 J albino mice (n = 11–12/group) were implanted SC in the right flank with B16-OVA tumors on Day 0. On Days 8 and 15, mice were dosed IT with 0.4 μg MEDI9197 or Vehicle, and dosed IP with 25 mg/kg GITRL FP or 12 mg/kg OX40 Ab. **a** Tumor volume as a mean (with last observation carried forward) ± SEM up to Day 43 and (**b**) spider plots of individual tumor volume. Statistical analysis was performed using two-way ANOVA with Tukey’s multiple comparisons test. ***p = 0.0005, ****p = 0.0001

To examine the combination potential of MEDI9197 with T cell co-stimulatory molecules, an OX40 agonist mAb or GITRL fusion protein (FP) was combined with a suboptimal dose of MEDI9197 in the B16-OVA model. While neither the OX40 agonist mAb, GITRL FP, nor MEDI9197 was efficacious as a monotherapy, significant tumor growth inhibition was observed when combining MEDI9197 administered IT with systemic administration of an anti-OX40 agonist (p = 0.005; compared with anti-OX40 alone) or a GITRL FP agonist (p ≤ 0.0001; compared with GITRL FP alone) (Fig. 7).

## Discussion

We report here that MEDI9197 is a TLR7/8 agonist driving robust activation of human adaptive and innate immune cells including both mDC and pDC. Like TLR7/8 agonists, TLR9 agonists and STING agonists delivered IT also promote anti-tumor immunity in mouse models [19, 30], and are currently being investigated in clinical trials (NCT02675439, NCT03172936, NCT02254772). However, we report here that these agonists have different effects on human immune cells. In particular, MEDI9197 activates human immune cells to secrete IFNα, IL-12 and IFNγ, whereas TLR9 and STING agonists only induced IFNα from human PBMC. Together this broader cytokine profile may be more effective at enhancing CD8^+^ T cell responses whilst inhibiting MDSC and Treg cells. Note that TLR9 is not expressed in human monocytes and myeloid dendritic cells, unlike TLR7 and TLR8 [31], which in part may explain the reduced ability of TLR9 ligands to enhance CD8 T cell responses in humans [32].

Despite the potent anti-tumor activity of systemic TLR agonists in pre-clinical models, clinical development of these agents has been hampered by the induction of CRS, and a lack of efficacy at tolerated doses [4, 10, 11, 33–38]. We report here that the unique structure and formulation of MEDI9197 enables it to be retained within the tumor after IT delivery, minimising systemic drug exposure and cytokine release, and driving sustained local TLR7/8 activation in the TME. It is likely that the limited changes in circulating TNFα cytokine levels observed following local MEDI9197 treatment were due to “spill over” of cytokines released from local immune cells at the injection site, since no systemic TNFα gene expression was detected in the spleen. Furthermore, retention and prolonged immune activation in the tumor by MEDI9197 appear critical for its anti-tumor activity, since IT delivery of Resiquimod, which rapidly disseminates and leads to systemic immune activation, failed to drive anti-tumor activity. Similarly, SC delivery of MEDI9197 at a site distal to the tumor was ineffective. The variability in dose retention (such as local dissemination at the treatment site) and the local immune milieu at the injection site may account for the variability observed in response to MEDI9197.

Positive responses to ICB therapy in patients has been shown to correlate with CD8^+^ T cell infiltration [3]. Tumors with low T cell infiltrate represent a significant unmet need. We report that activation of TLR7/8 in the tumor results in sustained transformation of the TME. In particular, MEDI9197 induced increased immune infiltration and the formation of ectopic lymph node structures in B16-OVA tumors. Type I IFN response genes were upregulated, CD8^+^ T cell infiltration was enhanced, and these cells were activated, expressing IFNγ. These data showing increased presence of CD8^+^ T cells and IFNγ suggest MEDI9197 IT delivery induces TME conversion to a hot immune phenotype. A similar inflammatory influx also correlated with tumor regression in response to topical Aldara (5% Imiquimod cream) in patients [39]. We have also shown increased NK cell killing of target cells and increased expression of CD69 on NK cells from mouse TILs, suggesting a role for NK cells in the anti-tumor activity of MEDI9197, which warrants further investigation.

The ability of MEDI9197 treatment to convert tumors from ‘cold’ to ‘hot’ make it an attractive co-therapy for ICB. PD-1, PD-L1, and CTLA4 and co-stimulatory molecules CD40, GITR, and OX40 are potential targets since MEDI9197 upregulates their expression in the TME. Our results confirm previous work demonstrating enhanced anti-tumor immunity in preclinical mouse models to the combination of MEDI9197 with PD-L1 blocking antibodies [23]. Furthermore, we go on to show combining MEDI9197 with PD-L1 blockade enhanced IFNγ production in a human DC/T cell MLR assay strengthening the rationale for combining MEDI9197 with ICB therapies targeting PD-1/PD-L1 interactions. The combination of MEDI9197 with GITRL FP or OX40 mAb also enhanced anti-tumor activity in the B16-OVA model. Studies in additional syngeneic models might provide insight into how different TMEs would impact on the activity of these combinations. These data are in agreement with the recent findings of Sagiv-Barfi et al [40] that in situ vaccination of an OX40 agonist with either TLR9 agonist SD-101 or Resiquimod resulted in an enhanced systemic anti-tumor immune response. Similar to our observations with MEDI9197, they also observe an increase in OX40 expression after intratumoral injection of SD-101. Beyond T cell-targeted therapies, others have also reported that MEDI9197 combination with CpG ODN enhanced antitumor immunity in mouse models. [24]. In addition, combinations of MEDI9197 with standard of care treatments for cancer, such as chemotherapy and radiotherapy, that drive release of tumor antigens are attractive given the observed vaccine adjuvant activity of MEDI9197 [22, 41–43]. Indeed, TLR7 agonists have previously been reported to enhance efficacy in combination with radiotherapy and chemotherapy in syngeneic mouse tumor models [44, 45].

## Conclusions

MEDI9197 is a TLR7/8 agonist that promotes robust and broad activation of human immune cells. MEDI9197 is uniquely retained at or near the site of injection to cause prolonged immune activation within the TME and in the local draining lymph nodes. IT injection of MEDI9197 results in tumor regression and enhanced survival in multiple mouse tumor models. IT therapy with MEDI9197 dramatically alters the TME by increasing CD8^+^ T cell infiltration and activation, increasing anti-tumor cytokines, and upregulating immune checkpoint expression. Combining MEDI9197 with other immune-modulatory agents can enhance anti-tumor activity, suggesting several routes to explore the utility of MEDI9197 in the clinic. MEDI9197 has been evaluated in Phase I clinical trials.

## Additional file

Additional file 1: Supplementary Information Materials and Methods. **Figure S1** In vitro characterisation of MEDI9197. **Figure S2** Rat serum MEDI9197 levels following SC or IM administration. **Figure S3** Local versus systemic cytokine induction following MEDI9197 administration in rodents. **Figure S4** Intratumoral administration is required for MEDI9197 anti-tumor effects. **Figure S5** IT administration of MEDI9197 modifies the tumor immune gene profile. **Figure S6** MEDI9197 enhances NK activation and gating strategies. (DOCX 1080 kb)

## Data Availability

The datasets used and/or analysed during the current study are available from the corresponding author on reasonable request.

## Abbreviations

APC: Antigen presenting cell
CRS: Cytokine release syndrome
dLN: Axilliary and brachial lymph nodes
FP: Fusion protein
ICB: Immune checkpoint blockade
IM: Intramuscular
IP: Intraperitoneal
IT: Intratumoral
mAbs: Monoclonal antibodies
mDC: Myeloid dendritic cells
MEC: Minimum effective concentration
MLR: Mixed lymphocyte reaction
mo-DC: Monocyte-derived DC
NK: Natural killer
PD: Pharmacodynamic
pDC: Plasmacytoid dendritic cells
QRA: Quantitative radiochemical analysis
QWBA: Quantitative whole-body autoradiography
SC: Subcutaneous
STING: Stimulator of interferon genes
TILs: Tumor infliltrating lymphocytes
TLR: Toll-like receptor
TME: Tumor microenvironment
