# Supplementary material for: Intratumoral immunotherapy with TLR7/8 agonist MEDI9197 modulates the tumor microenvironment leading to enhanced activity when combined with other immunotherapies
Source: J Immunother Cancer. 2019 Sep 11;7:244. doi: 10.1186/s40425-019-0724-8 (PMC6739946; doi:10.1186/s40425-019-0724-8)

Additional file 1

**Supplementary Information Materials and Methods**

| **Media Recipes** | |
| --- | --- |
| **Assay (media name)** | **Recipe** |
| **TLR reporter cell assay (complete media)** | **DMEM with 4.5 g/L glucose, 10% fetal bovine serum, 2 mM glutamine 50 U/ml penicillin, 50 µg/ml streptomycin, 100 µg/ml Normocin (InvivoGen), 30 µg/ml Blasticidin (InvivoGen), and 100 µg/ml Zeocin (InvivoGen),** |
| **PBMC activation (PBMC media)** | **RPMI Medium 1640+GlutaMAX (Gibco), 25 mM HEPES (Gibco), 10% (v/v) FBS (Gibco), 1% (v/v) Penicillin/Streptomycin (Gibco).** |
| **gene expression in monocyte derived macrophages (complete media for monocyte derived macrophages)** | **RPMI Medium 1640+GlutaMAX (Gibco) supplemented with 10% (v/v) FBS (Gibco) and 1% (v/v) Penicillin/Streptomycin (Gibco).** |
| **PHA-induced cytokine production assay (complete media)** | **RPMI Medium 1640+GlutaMAX (Gibco), 25 mM HEPES (Gibco), 10% (v/v) FBS (Gibco), 1% (v/v) Penicillin/Streptomycin (Gibco)** |
| **NK cell killing assay and peptide-specific killing an recall assay** | **RPMI Medium 1640+GlutaMAX (Gibco), 5% (v/v) human AB serum (Sigma), 1% (v/v) Penicillin/Streptomycin (Gibco)** |
| **mouse splenocyte cytokine production (splenocyte media)** | **RPMI Medium 1640+GlutaMAX (Gibco), 5% (v/v) FBS (Gibco), 1% (v/v) Penicillin/Streptomycin (Gibco).** |
| **B16-OVA cell line** | **Dulbecco's Modified Eagle Medium (DMEM) with 10% heat-inactivated fetal calf serum, 0.5 mg/mL of Penicillin/Streptomycin, and G418 sulfate at 1 mg/mL (Thermo Fisher Scientific)** |
| **B16-F10 AP3 CAG luc2 cell line** | **miminum essential medium (MEM) with 10% fetal bovine serum, 1% non-essential amino acids, 1% sodium pyruvate, and 0.7 µg/mL puromycin** |
| **4T1 and MC38 cell lines** | ***Mosely et al, Cancer Immunol Res, 2017*** |

| **Antibodies for Flow Cytometry** | | | |
| --- | --- | --- | --- |
| **Antigen** | **Clone** | **Company** | **Figure** |
| CD16 | 3G8 | BD | Fig 1D |
| CD25 | 2A3 | BD | Fig 1D |
| CD123 | 7G3 | BD | Fig 1D |
| HLA-DR | G46-6 | BD | Fig 1D |
| CD3 | SK7 | Biolegend | Fig 1D |
| CD14 | HCD14 | Biolegend | Fig 1D |
| CD19 | HIB19 | Biolegend | Fig 1D |
| CD20 | 2H7 | Biolegend | Fig 1D |
| CD40 | 5C3 | Biolegend | Fig 1D |
| CD56 | MEM-188 | Biolegend | Fig 1D |
| CD56 | HCD56 | Biolegend | Fig 1D |
| CD80 | 2D10 | Biolegend | Fig 1D |
| BDCA4 | AD5-17F6 | Miltenyi Biotec | Fig 1D |
| CD3 | UCHT1 | Biolegend | Fig 1H and I |
| CD8 | LT8 | Proimmune | Fig 1H and I |
| granzyme B | GB11 | eBioscience | Fig 1H and I |
| CD3ε | 17A2 | Miltenyi Biotec | Fig 5C-D and Supp Fig 5A |
| CD8α | 53-6.7 | Miltenyi Biotec | Fig 5C-D and Supp Fig 5A |
| CD4 | REA604 | Miltenyi Biotec | Fig 5C-D and Supp Fig 5A |
| CD45 | 30F11 | Miltenyi Biotec | Fig 5C-F, 6 and Supp Fig 5A |
| CD69 | H1.2F3 | Miltenyi Biotec | Fig 5C-D and Supp Fig 5A |
| CD335 | 29A1.4 | ThermoFisher | Fig 5C-D and Supp Fig 5A |
| rat IgG2a |  | ThermoFisher | Fig 5C-D and Supp Fig 5A |
| CD3ε | 500A2 | eBioscience | Fig 5F |
| CD8α | 53-6.7 | ThermoFisher | Fig 5F |
| IFNγ | XMG1.2 | ThermoFisher | Fig 5F |
| TNFα | MP6-XT22 | ThermoFisher | Fig 5F |
| IL-2 | JES6-5H4 | ThermoFisher | Fig 5F |
| syrian hamster IgG |  | ThermoFisher | Fig 5F |
| rat IgG1k |  | ThermoFisher | Fig 5F |
| rat IgG1k |  | ThermoFisher | Fig 5F |
| rat IgG2b |  | ThermoFisher | Fig 5F |
| TCRβ | H57-597 | Biolegend | Fig 5E and 6 |
| CD8α | 53-6.7 | ThermoFisher | Fig 5E and 7 |
| CD44 | IM7.8.1 | Miltenyi Biotec | Fig 5E and 8 |
| CD62L | ERA828 | Miltenyi Biotec | Fig 5E and 9 |
| PD-1 | J43 | ThermoFisher | Fig 6 |
| PD-L1 | 10F.9G2 | ThermoFisher | Fig 7 |

| **Taqman probes** | | | |
| --- | --- | --- | --- |
| **gene name** | **assay number** | **Company** | **Figure** |
| CD80 | Hs01045161_m1 | Thermofisher | Supp Fig 1E |
| CD274 | Hs00204257_m1 | Thermofisher | Supp Fig 1E |
| CD40 | Hs01002915_g1 | Thermofisher | Supp Fig 1E |
| IL12A | Hs01073447_m1 | Thermofisher | Supp Fig 1E |
| GAPDH | Hs02786624_g1 | Thermofisher | Supp Fig 1E |
| Gapdh | Mm99999915 g1 | applied biosystems | Supp Fig 3 |
| Tnf | Mm00443258 m1 | applied biosystems | Supp Fig 3 |
| Il12p40 | Mm00434174 m1 | applied biosystems | Supp Fig 3 |
| Ifng | Mm00801778_m1 | applied biosystems | Supp Fig 3 |
| Mx1 | Mm00487796_m1 | applied biosystems | Supp Fig 3 |
| Oas2 | Mm00460961_m1 | applied biosystems | Supp Fig 3 |
| Tnfsf10 | Mm00437174_m1 | applied biosystems | Supp Fig 3 |
| GusB | Mm.3317 | Qiagen | Fig 5B, 6A-B |
| Fasl | Mm.3355 | Qiagen | Fig 5B |
| Gzmb | Mm.14874 | Qiagen | Fig 5B |
| Ifng | Mm.240327 | Qiagen | Fig 5B |
| Tnf | Mm.1293 | Qiagen | Fig 5B |
| Cd3e | Mm.210361 | Qiagen | Fig 5B |
| Cd8a | Mm.1858 | Qiagen | Fig 5B |
| Cxcl10 | Mm.877 | Qiagen | Fig 5B |
| Ido1 | Mm.392 | Qiagen | Fig 6A and Supp Fig 5 |
| Ido2 | Mm.219580 | Qiagen | Fig 6A and Supp Fig 5 |
| Pdcd1 | Mm.5024 | Qiagen | Fig 6A and Supp Fig 5 |
| Cd274 | Mm.245363 | Qiagen | Fig 6A and Supp Fig 5 |
| Ctla4 | Mm.390 | Qiagen | Fig 6A and Supp Fig 5 |
| Cd40 | Mm.271833 | Qiagen | Fig 6B and Supp Fig 5 |
| Tnfrsf4 | Mm.13885 | Qiagen | Fig 6B and Supp Fig 5 |
| Tnfrsf18 | Mm.3180 | Qiagen | Fig 6B and Supp Fig 5 |
| Tnfrsf9 | Mm.244187 | Qiagen | Fig 6B and Supp Fig 5 |
| B2m | Mm.163 | Qiagen | Supp Fig 5 |
| Il1b | Mm.222830 | Qiagen | Supp Fig 5 |
| Mki67 | Mm.4078 | Qiagen | Supp Fig 5 |
| ActB | Mm.328431 | Qiagen | Supp Fig 5 |
| Prf1 | Mm.240313 | Qiagen | Supp Fig 5 |
| Ifnb | Mm.1245 | Qiagen | Supp Fig 5 |
| Timp1 | Mm.8245 | Qiagen | Supp Fig 5 |
| Il27 | Mm.222632 | Qiagen | Supp Fig 5 |
| Tlr7 | Mm.23979 | Qiagen | Supp Fig 5 |
| Ccl8 | Mm.42029 | Qiagen | Supp Fig 5 |
| Il10 | Mm.874 | Qiagen | Supp Fig 5 |
| Arg1 | Mm.154144 | Qiagen | Supp Fig 5 |
| IfnI3 | Mm.246799 | Qiagen | Supp Fig 5 |
| Timp2 | Mm.206505 | Qiagen | Supp Fig 5 |
| Il6 | Mm.1019 | Qiagen | Supp Fig 5 |
| Tlr8 | Mm.196676 | Qiagen | Supp Fig 5 |
| Ifna1 | Mm.57127 | Qiagen | Supp Fig 5 |
| Il15 | Mm.4392 | Qiagen | Supp Fig 5 |
| Cxcl2 | Mm.4979 | Qiagen | Supp Fig 5 |
| Il2 | Mm.14190 | Qiagen | Supp Fig 5 |
| Cxcl9 | Mm.766 | Qiagen | Supp Fig 5 |
| Ccl5 | Mm.284248 | Qiagen | Supp Fig 5 |
| Batf3 | Mm.6922 | Qiagen | Supp Fig 5 |
| FoxP3 | Mm.182291 | Qiagen | Supp Fig 5 |
| Nos2 | Mm.2893 | Qiagen | Supp Fig 5 |
| Tbx21 | Mm.94519 | Qiagen | Supp Fig 5 |
| MX1 | Mm.PT.58.12101853.g | Integrated DNA technologies | Fig 5A |
| Isg15 | Mm.PT.58.41476392.g | Integrated DNA technologies | Fig 5A |
| Ifit1 | Mm.PT.58.32674307 | Integrated DNA technologies | Fig 5A |
| Ifit3 | Mm.PT.58.33537107 | Integrated DNA technologies | Fig 5A |
| GusB | Mm.PT.39a.22214848 | Integrated DNA technologies | Fig 5A |

*Mo-DC activation and cytokine production*

Human Mo-DC were prepared from human peripheral blood by density gradient centrifugation (Histopaque), and monocytes were enriched by adherence to tissue culture plates. Monocytes were differentiated into DCs by culturing the cells in 1000 U/mL GM-CSF and 1000 U/mL IL-4 for 5 days. The Mo-DC were cultured in AIM V serum-free medium supplemented with 1000 U/mL IFNγ ± MEDI9197, Vehicle (DMSO), or Medium alone at 5×10^5^ cells/well. After 24 hr, IL-12p70 levels were measured in the supernatant by ELISA.

*Measurement of gene expression in primary human monocyte-derived macrophages*

For RNA analysis, cells treated for 8h were lysed using the MagMAX 96 Total RNA isolation Kit (ThermoFisher) and RNA was isolated according to the manufacturer’s instructions. Complementary DNA was generated using the High capacity cDNA Reverse Transcription Kit (ThermoFisher) following the manufacturer’s protocol. qRT-PCR analysis was performed using the following commercially available TaqMan® gene expression assays: CD80 FAM/MGB, CD274 FAM/MGB, CD40 FAM/MGB, IL12A FAM/MGB and GAPDH VIC/TAMRA and the Taqman Fast Advanced Master Mix (ThermoFisher). The reactions were run on the [QuantStudio 12K Flex Real-Time PCR System](https://www.thermofisher.com/uk/en/home/life-science/pcr/real-time-pcr/real-time-pcr-instruments/quantstudio-12k-flex-real-time-pcr-system.html) (ThermoFisher) using the following program: one cycle of 95°C for 20 sec, 40 cycles of 95°C for 1 second, and 60°C for 20 sec. Target gene expression was normalised to housekeeping gene (*GAPDH*) expression in each sample (ΔC_T_ = Target gene C_T_ - GAPDH C_T_) and then to the target gene expression in untreated samples (ΔΔC_T_ = ΔC_T_(treated) - ΔC_T_(untreated). Finally, the data was converted using the =2^-ΔΔC_T_ formula to derive fold change in target gene expression compared to the untreated.

*Peptide-specific killing and recall assay*

For flow cytometry, cells were first blocked with human Trustain X Fc block solution (Biolegend), then stained for CMV peptide for 30 min (pp65 pentamer, Proimmune), followed by a Blue/UV viability stain (Invitrogen). After fixation with the Foxp3 / Transcription Factor Staining Buffer Set (eBioscience), granzyme B was stained intracellularly.

*Human PBMC and mouse splenocyte viability assay*

Supernatants were discarded, and fresh cell culture medium was added. Viability was quantified using the CellTiter-Glo 2.0 Assay (Promega) according to manufacturer’s instructions.

*Mouse splenocyte cytokine production*

Splenocytes from C57BL/6 mice were cultured for 24 hr at 37°C with MEDI9197, Resiquimod, Imiquimod or DMSO. Mouse IFN-γ in supernatants was measured by MSD (Meso Scale Diagnostics, MSD V-plex mouse IFN-γ) according to the manufacturer’s instructions.

*MEDI9197 quantitation from rat serum*

Serum was prepared by collecting blood via the jugular and vena cava veins, except for the terminal bleed, which was collected by heart puncture. MEDI9197 serum levels were measured by liquid chromatography-tandem mass spectrometry (LC-MS/MS). The LLQ for serum MEDI9197 is 0.5 ng/mL (0.84 pmol/mL).

*Measurement of gene expression in mouse lymph nodes and spleens following S.C. injection of MEDI9197 and Resiquimod*

Reagents used for total RNA purification, 1^st^ strand cDNA synthesis, and qPCR include: RNeasy Mini Kit, QIAshredder, RNase-free DNase Set, QuantiTect Rev. Transcription Kit, QuantiTect Probe PCR Kit were obtained from Qiagen.

MEDI9197, Resiquimod, and Vehicle formulations were prepared in an oil-in-water (O/W) emulsion as described in ‘In vivo *studies’* section of Materials and Methods in manuscript.

Axial and brachial lymph nodes, along with spleens from each treatment group were pooled and stored in RNAlater Solution (ThermoFisher Scientific). After tissue was collected following the last time point, total RNA was isolated using RNeasy Mini Kit according to the manufacturer’s protocol (Qiagen). For spleens, sections weighing approximately 10 mg were cut, blotted to eliminate excess of RNAlater solution, and disassociated using KONTES RNase-Free Pellet Pestle Grinders from Kimble Chase in 0.6 mL lysis buffer (RLT with β-2 Mercaptoethanol, Qiagen). Lysates were cleared using QIAshredder (Qiagen), and then one volume of 70% ethanol was added to each lysate and samples were loaded onto RNeasy columns and centrifuged according to manufacturer’s recommendation. Samples were washed several times with washing buffers. Total RNA was treated with DNase1 to remove genomic DNA, washed again with RPE buffer, and eluted with RNase-free water (ThermoFisher Scientific).

Conditions for the reverse transcriptase reaction and generation of 1^st^ strand cDNA were as described in manuals for QuantiTect Reverse Transcription and QuantiTect Probe PCR kits (Qiagen). In preparation for qPCR, 1 uL of cDNA was diluted in 14 µL of RT-PCR grade water. Each 20 µL PCR reaction contained 10 µL of 2x QuantiTect Probe PCR Master Mix, 1 µL of gene specific 20x TaqMan Gene Expression Assay primers, 2 µL of diluted cDNA, and 7 µL of RT-PCR grade water. A step program for Q-PCR was used as follows: one cycle of 50^◦^C for 2 min, one cycle of 95^◦^C for 10 min, 45 cycles of 95^◦^C for 15 seconds, and 50^◦^C for 1 min.

Relative quantitation of gene expression was performed using the comparative cycle threshold (Ct) method. Real Time qRT-PCR data were analyzed with Applied Biosystem’s SDS version 2.3 software. The expression level of the GAPDH gene was used for normalization of data, and the relative expression levels were quantified using the ΔCt Method, which is a variation of the Livak method (Livak, 2001, Methods, 25:402). The results presented are from pooled samples (n=3 draining lymph nodes (DLNs) or 3 spleens). In brief, Ct values for the reference gene (GAPDH) and target genes (cytokines and cytokine-inducible genes) in each sample were determined. Then the Ct values for the target genes were normalized to the reference gene by calculating the ΔCt values (GAPDH Ct – Target gene Ct), and relative gene expression for each sample was determined using the following formula:

Relative Quantitation = 2^ΔCt^ = 2^(GAPDH Ct – Cytokine or Cytokine-inducible gene CT)^

Last, fold change between treated (MEDI9197, Resiquimod, Vehicle) relative to the untreated group was calculated as follows:

Fold-Change = Treated 2^ΔCt^ ÷ Untreated 2^ΔCt^ = Relative Quantitation of Treated (MEDI9197, Resiquimod, Vehicle) ÷ Relative Quantitation of Untreated

*MSI analysis supplementary methods*

Analytical grade acetonitrile, methanol and formic acid were obtained from Fisher Scientific. Dihydroxybenzoic acid was obtained from Sigma Aldrich.

In order to minimize any potential degradation of the drug and any endogenous compounds, tumors were embedded in plastic molds (VWR International Ltd) using 10% w/v Poly[N-(2-hydroxypropyl)methacrylamide] (pHPMA) solution. pHPMA blocks were stored at -80ºC prior to cryosectioning.

pHPMA embedded tissues were cryosectioned on a CM3050S cryomicrotome (Leica Biosystems) at a thickness of 10 μm and thaw mounted onto indium tin oxide (ITO) coated MALDI target slides (Bruker Daltonics). Thaw-mounted slides were immediately desiccated using a stream of dry N_2_ prior to vacuum packing and storage at −80°C until analysis.

Adjacent sections were sectioned onto Superfrost glass slides (Fisher Scientific) for subsequent DESI-MSI analysis if needed. Tissue sections were taken at approximately equal depth from all samples.

Vacuum packed, thaw mounted tissue sections were allowed to reach room temperature after removal from -80^o^C storage, prior to breaking the vacuum seal. Optical images were taken using a standard flat-bed scanner (Seiko Epson) prior to MALDI matrix application. Matrix coating was applied using a TMsprayer (HTX technologies) set at 75°C and performing 8 passes, with a back-up flow of 50% methanol/water *v/v* at a flow rate of 0.08 mL/min and nebulized with nitrogen at 8 psi. The matrix used for the analysis of MEDI9197 was 2,4-Dihydroxybenzoic acid (35 mg/mL, 50/50/0.1 *v/v/v* acetonitrile/water/trifluoroacetic acid).

**Supplementary Information Figure Legends**

**Figure S1. In vitro characterisation of MEDI9197.**

**A,** Viability (CellTiter-Glo® 2.0 Assay) of human PBMCs (left) or C57BL/6 mouse splenocytes (right) after 24hrs of culture with a titration of MEDI9197, Resiquimod, or Imiquimod. Data are luminescence signal normalised to DMSO control. Data show the mean of technical triplicates ± SEM for 6 (human) or 9 (mouse) biological replicates and are representative of 2 (human) or 3 (mouse) independent experiments. **B,** Secreted embryonic alkaline phosphate (SEAP) reporter activity in HEK293-NFκB-SEAP cells expressing mouse TLR7. Cells were cultured for 24 hr with MEDI9197 at various concentrations. Results are shown as fold-change relative to Vehicle control and are representative for 3 individual experiments. **C,** Release of IFN-γ from splenocytes of C57BL/6 mice, stimulated for 24 hrs with a titration of MEDI9197, Resiquimod, Imiquimod or DMSO Vehicle control. IFN-γ was measured in supernatants from treated splenocytes using a MSD V-plex assay for mouse IFN-γ. Data show the mean of technical triplicates ± SEM and is representative for 9 biological replicates of 3 independent experiments. **D**, IL-12p70 secretion was evaluated from MEDI9197-stimulated human Mo-DC. Mo-DC were generated from adherent PBMC cultured for 5 days with recombinant GM-CSF and recombinant IL-4. Non-adherent Mo-DC were cultured with 1000 U/mL recombinant IFN-γ and treated with various concentrations of MEDI9197, DMSO Vehicle, or left untreated (Medium) for 24 hr. Secreted IL-12p70 was measured from the supernatants by ELISA. The results are shown as mean, SEM, n=5 donors from 5 separate experiments. **E,** CD14^+^ PBMCs were differentiated into monocyte derived macrophages by culturing with M-CSF for 6 days. Cells were then primed for 24 hrs with IFN-γ, followed by an 8-hour incubation with either 20 ng/ml LPS or 3 µM MEDI9197. Cells were lysed for RNA isolation and amplification of indicated genes. Target gene expression was normalised to housekeeping gene (*GAPDH*) expression in each sample (ΔC_T_ = Target gene C_T_ - GAPDH C_T_) and then to the target gene expression in untreated samples (ΔΔC_T_ = ΔC_T_(treated) - ΔC_T_(untreated). The data was converted using the =2^-ΔΔC_T_ formula to derive fold change in target gene expression compared to the untreated. Data show the mean of 5 donors and is representative of 2 individual experiments. *p<0.05, **p<0.01,****p<0.0001

**Figure S2. Rat serum MEDI9197 levels following SC or IM administration.**

Rat serum MEDI9197 levels were quantified by LC-MS/MS 0 hr and 2-24 hrs after a single (**A)** SC or (**B)** IM dose of 0.1, 0.3, and 1 mg/kg MEDI9197. Data are shown as mean ng/mL, SD. The values at the 0 hr time point are an average of 30 rats (1 serum sample per rat). The values shown at all other time points are an average of 5 rats (1 serum sample per rat). The LLQ for serum MEDI9197 is 0.5 ng/ml (0.84 pmol/mL). MEC is the minimum effective concentration required to induce cytokines in human PBMCs.

**Figure S3. Local versus systemic cytokine induction following MEDI9197 administration in rodents.**

Mouse proinflammatory cytokine (*Tnfα, Il-12p40, Ifnγ*) and type I IFN-inducible (*Mx1, Oas2, Tnfs10*) mRNA were measured in draining LNs and spleens following a single SC injection in the dorsoscapular region of MEDI9197 (20 µg), Resiquimod (20 µg), Vehicle. Some mice were untreated (Naïve). Draining LNs and spleens were collected 1, 3, 6, and 18 hr post-dose. The tissues were pooled from each group prior to RNA preparation and cDNA synthesis. qRT-PCR was used to determine the relative amount of cytokine mRNA to GAPDH mRNA. The results are expressed as the fold-change of each treated group (MEDI9197, Resiquimod, and Vehicle) compared to the Naive group (n=3 mice/group/time point).

**Figure S4. Intratumoral administration is required for MEDI9197 anti-tumor effects.**

**A,** Tumor growth was measured in the single-flank B16-OVA tumor model following IT injection or SC dosing away from the tumor on the opposite flank. C57BL/6J albino mice were implanted SC B16-OVA tumor cells the left flank on Day 0 (20 mice/group). On Days 8 and 15, mice were dosed IT with MEDI9197 [20 µg/50 µL], Resiquimod [20 µg/50 µL], or Vehicle (sesame oil/EtOH, 50 µL]). Some mice were dosed SC with MEDI9197 on the opposite side of the implanted tumor (right flank). Naïve mice were untreated. Mice were euthanized when tumor size equaled or exceeded 2500 mm^3^. Spider plots show tumor volume for all mice up to Day 90. **B-D**, Tumor growth was measured in the single-flank B16-OVA tumor model following one (**B** and **D**) or 2 (**C**) IT doses of MEDI9197. C57BL/6J albino mice were implanted SC B16-OVA tumour cells on the left flank. 8-9 days after cell implantation, mice were administered a single dose of MEDI9197 at 20 µg (**C**) and 4, 1, or 0.1 µg (**D**); or administered 2 doses 7 days apart at 20 or 100 µg (**B**). Average tumor volume is displayed with Gaussian non-linear fit ± SEM.

**Figure S5. IT administration of MEDI9197 modifies the tumor immune gene profile.**

Gene expression in B16-OVA tumors were measured from MEDI9197 responder and non-responder mice at 7 and 11 days post IT dose (20 µg MEDI9197 or Vehicle). Total RNA was isolated from each tumor and used for cDNA synthesis (n=5 per group per time point). RT-PCR was performed in duplicate to determine expression relative to control gene expression (GusB). The results indicate fold-change in gene expression relative to the Vehicle control group.  Heat map comparing the fold change in gene expression of individual responders compared to non-responders at days 7 and 11 post dosing.

**Figure S6. MEDI9197 enhances NK activation and gating strategies.**

**A**, The percent of NK cells (CD45^+^/CD3ε^-^/CD335^+^) in the CD45^+^ population and activated NK cells (CD69^+^) were quantified by flow cytometry from MEDI9197 (20 μg) and Vehicle injected tumors after 1, 7, and 11 days post-dose. Statistical analysis was performed by 2-way ANOVA with Sidak’s test. ****p=<0.0001, n=4 mice/treatment group/day **B**, gating strategy for CD69 expression on T cells. **C**, Gating strategy for PD-1/PD-L1 expression on CD8^+^ T cells and effector T cells. **D**, Gating strategy for intracellular cytokine expression on CD8^+^ T cells.


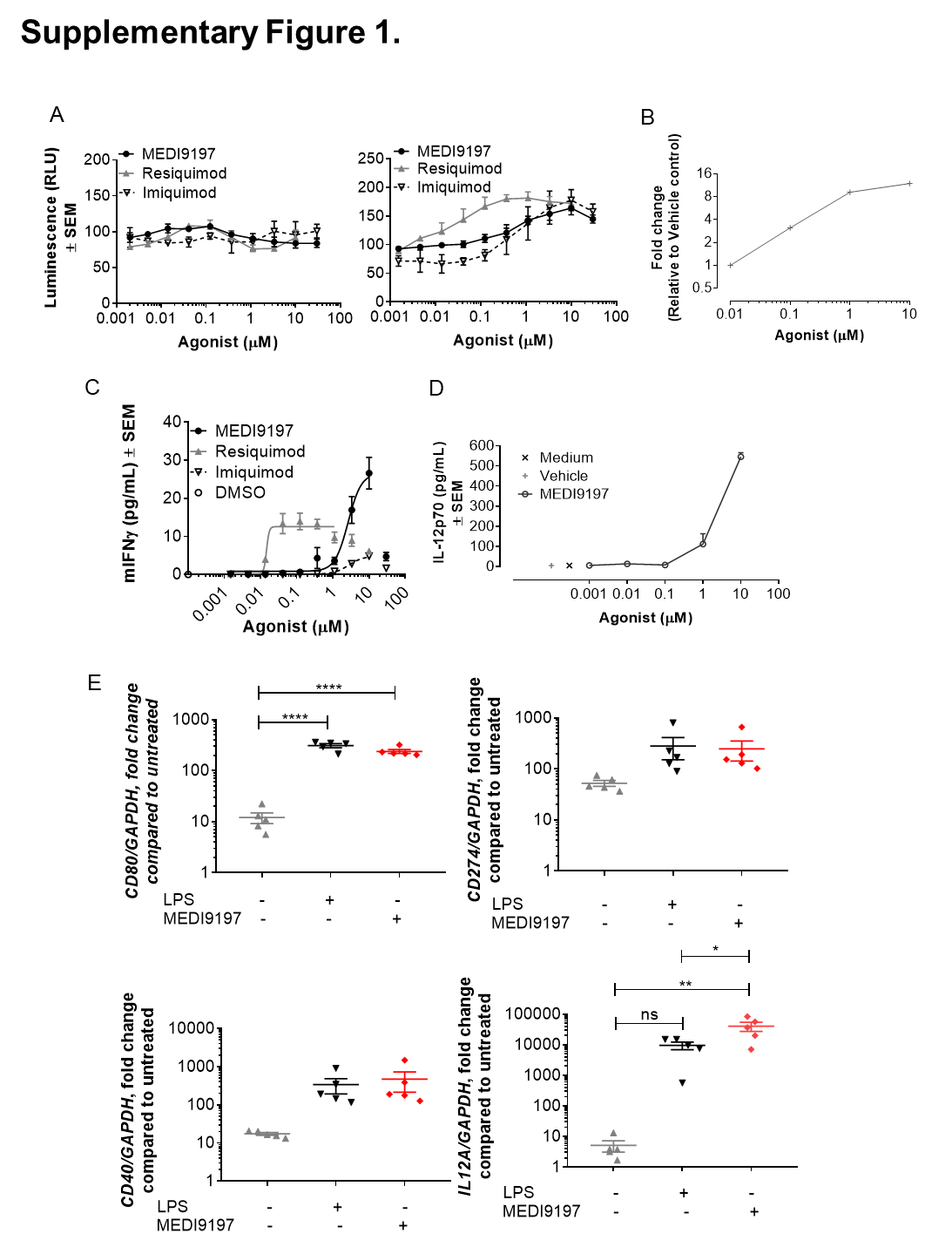


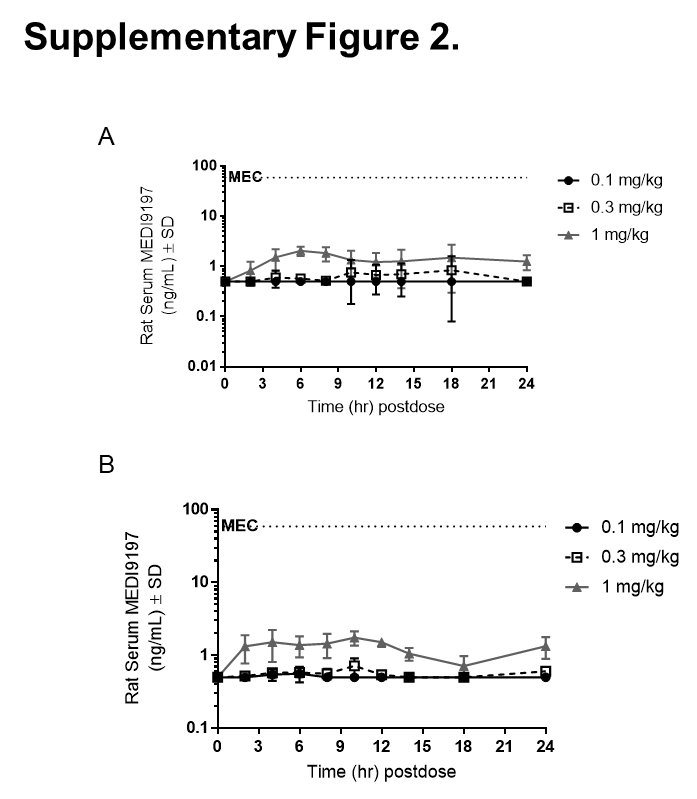


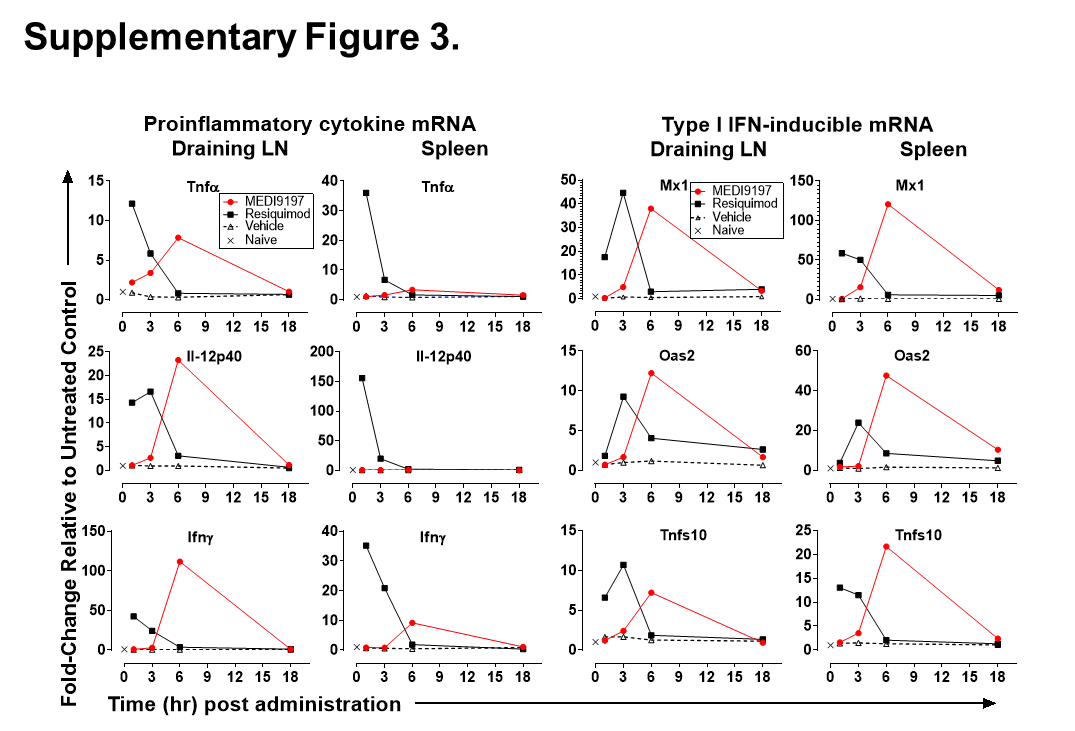


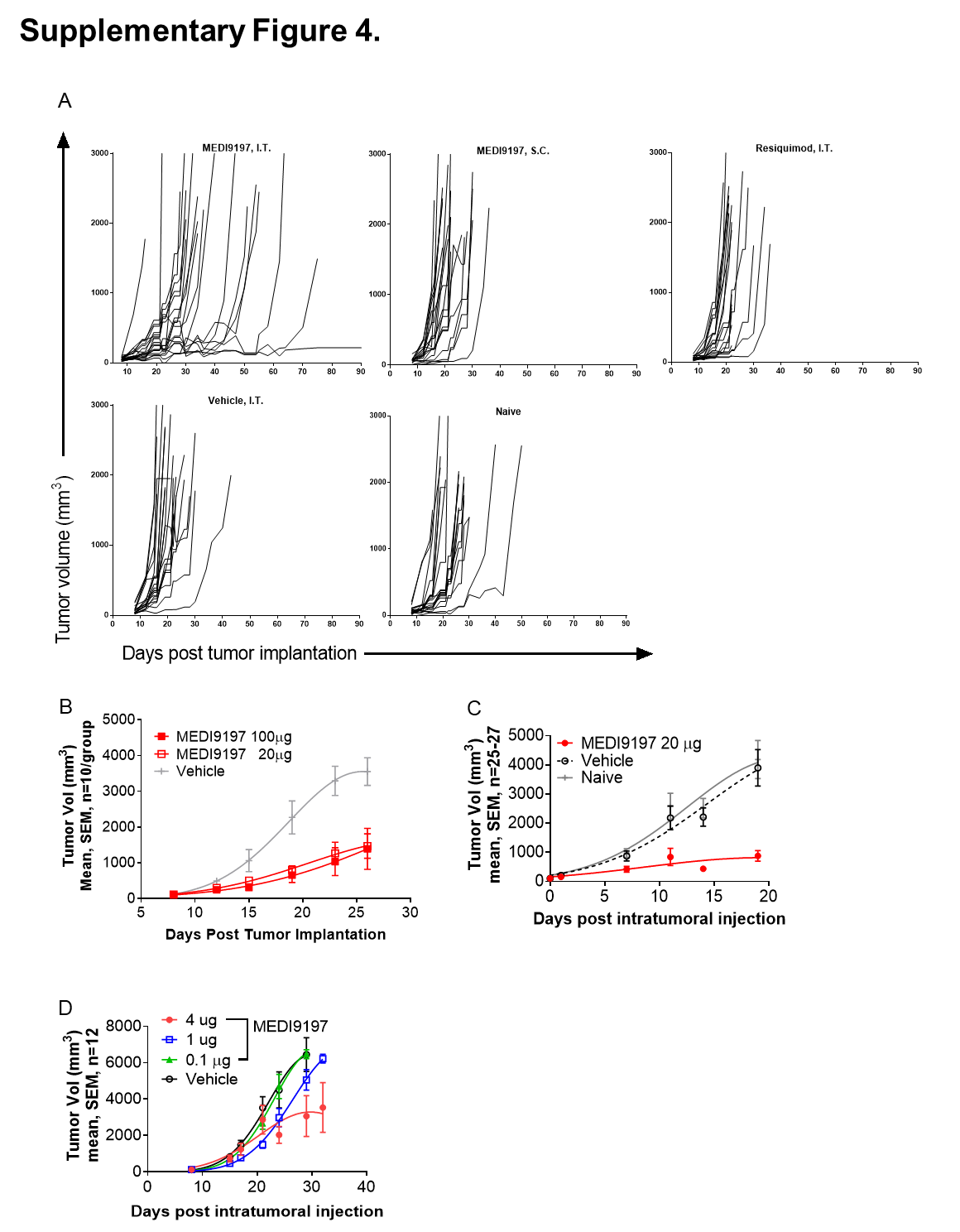


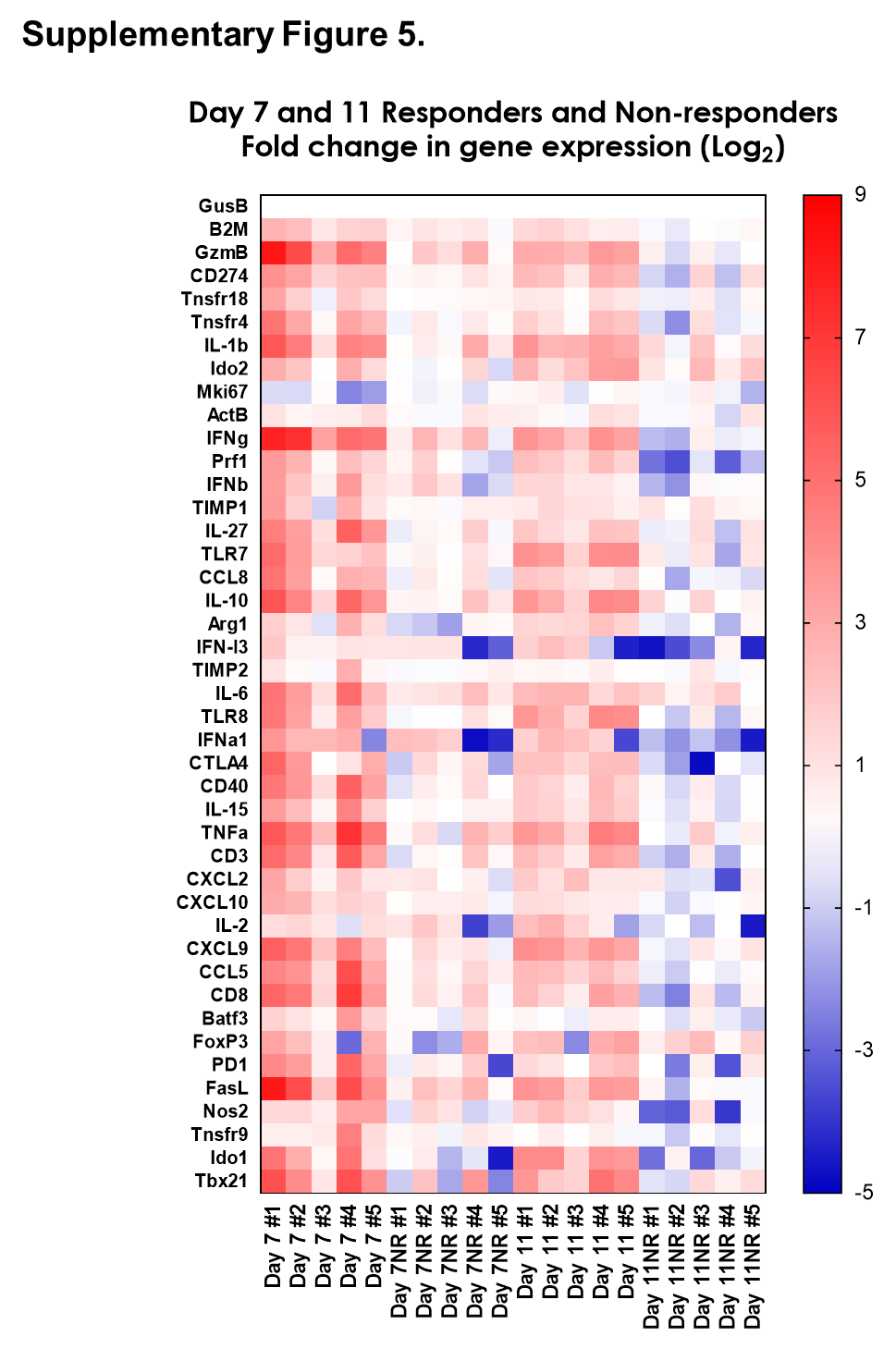


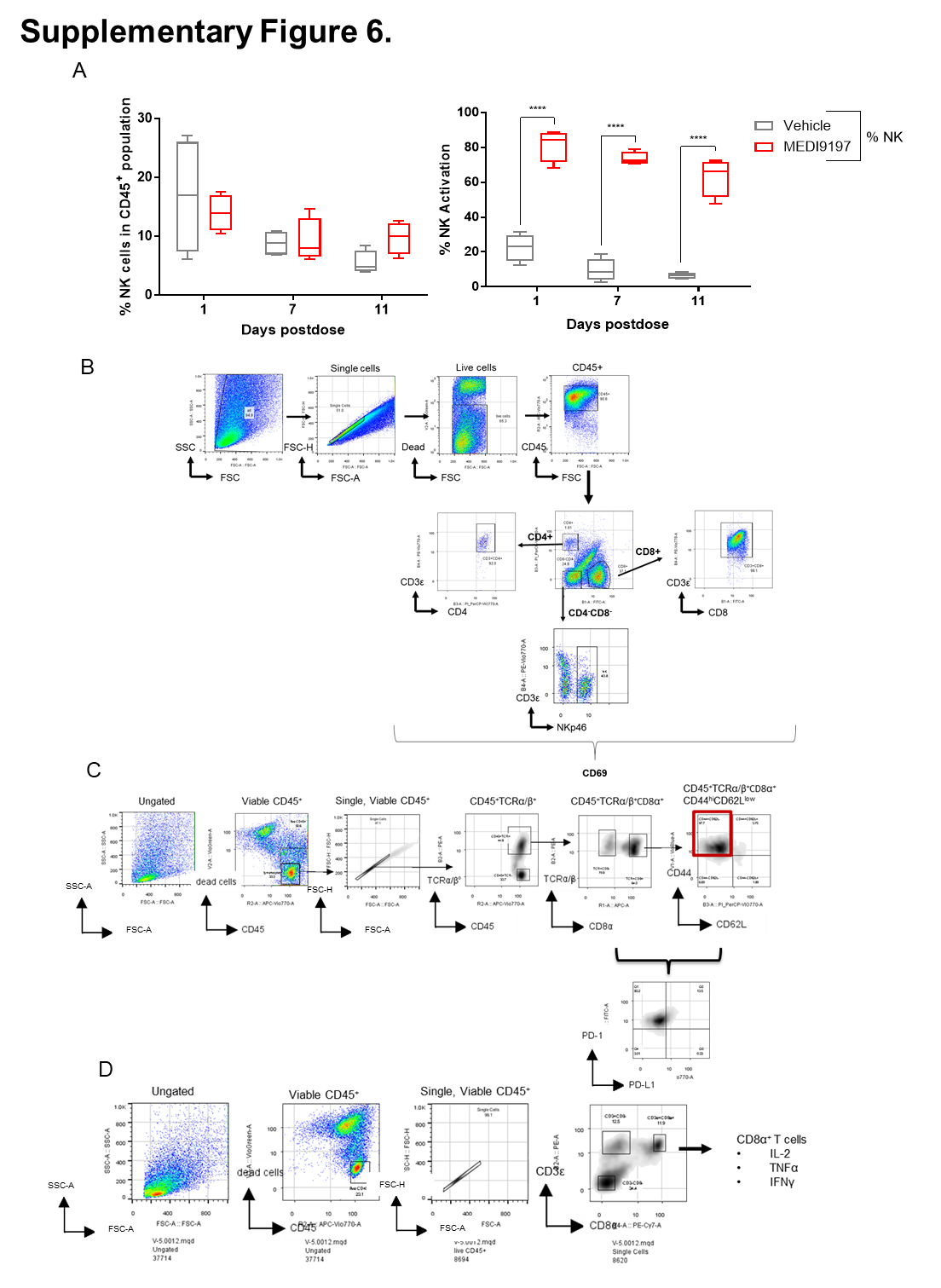

Supplement: Supplementary file 1 — Supplementary Information Materials and Methods. Figure S1 In vitro characterisation of MEDI9197. Figure S2 Rat serum MEDI9197 levels following SC or IM administration. Figure S3 Local versus systemic cytokine induction following MEDI9197 administration in rodents. Figure S4 Intratumoral administration is required for MEDI9197 anti-tumor effects. Figure S5 IT administration of MEDI9197 modifies the tumor immune gene profile. Figure S6 MEDI9197 enhances NK activation and gating strategies. (DOCX 1080 kb) [file 40425_2019_724_MOESM1_ESM.docx]
